# Pan‐genome: A promising resource for noncoding RNA discovery in plants

**DOI:** 10.1002/tpg2.20046

**Published:** 2020-08-28

**Authors:** Muhammad Tahir ul Qamar, Xitong Zhu, Muhammad Sarwar Khan, Feng Xing, Ling‐Ling Chen

**Affiliations:** ^1^ State Key Laboratory for Conservation and Utilization of Subtropical Agro‐bioresources College of Life Science and Technology Guangxi University Nanning 530004 P. R. China; ^2^ Hubei Key Laboratory of Agricultural Bioinformatics College of Informatics Huazhong Agricultural University Wuhan 430070 P. R. China; ^3^ Center of Agricultural Biochemistry and Biotechnology University of Agriculture Faisalabad 38000 Pakistan; ^4^ College of Life Science Xinyang Normal University Xinyang 464000 P. R. China

## Abstract

Plant genomes contain both protein‐coding and noncoding sequences including transposable elements (TEs) and noncoding RNAs (ncRNAs). The ncRNAs are recognized as important elements that play fundamental roles in the structural organization and function of plant genomes. Despite various hypotheses, TEs are believed to be a major precursor of ncRNAs. Transposable elements are also prime factors that cause genomic variation among members of a species. Hence, TEs pose a major challenge in the discovery and analysis of ncRNAs. With the increase in the number of sequenced plant genomes, it is now accepted that a single reference genome is insufficient to represent the complete genomic diversity and contents of a species, and exploring the pan‐genome of a species is critical. In this review, we summarize the recent progress in the field of plant pan‐genomes. We also discuss TEs and their roles in ncRNA biogenesis and present our perspectives on the application of pan‐genomes for the discovery of ncRNAs to fully explore and exploit their biological roles in plants.

**Abbreviations**
circRNA: circular RNA
CNV: copy number variant
GBS: genotyping‐by‐sequencing
GWAS: genome‐wide association study
lncRNA: long noncoding RNA
LTR: long terminal repeat
miRNA: microRNA
MITE: miniature inverted‐repeat transposable element
ML: machine learning
ncRNA: noncoding RNA
NGS: next‐generation sequencing
PAV: presence/absence variant
siRNA: small interfering RNA
SNP: single nucleotide polymorphism
SV: structural variation
TE: transposable element.

## 1 INTRODUCTION

In plants, a major portion of the genome is developmentally transcribed to produce ncRNAs and plays a crucial role in regulating many critical processes (Hou et al., 2019). The ncRNAs involved in regulatory processes are generally categorized into short ncRNAs, such as small interfering RNAs (siRNAs) and microRNAs (miRNAs), long ncRNAs (lncRNAs), and circular RNAs (circRNAs) (Wei, Huang, Yang, & Kang, 2017). Among the short ncRNAs, siRNAs (20–25 bp) play important roles in several processes including plant defense and stress responses (Borges et al., 2018; Martinez et al., 2018; Zhang et al., 2016), whereas miRNAs (∼22 nt) participate in a variety of crucial biological processes including metabolism, development, stress response, transcription factor regulation, and antisilencing (Bartel, 2004; Fahlgren et al., 2007; Li, Li, Xia, & Jin, 2011; Voinnet, 2009). By contrast, lncRNAs (usually >200 bp long) are involved in a variety of cellular functions and gene regulation or silencing pathways (Wierzbicki, Haag, & Pikaard, 2008). Additionally, circRNAs, new members of the ncRNA family, are ubiquitously expressed in plant genomes and play important roles in processes such as protein binding, miRNA binding, and transcriptional regulation (Zhao, Chu, & Jiao, 2019). Although a large number of ncRNAs have been identified to date, their true biological functions, mechanisms of origin, and role in genomic diversity remain largely unknown.

Advances in high‐throughput sequencing technologies over the last decade have led to innovative approaches for analyzing plant genome content, diversity, and evolution. Previously, genomic studies were commonly focused on a single reference genome using standard approaches, such as the low‐throughput and expensive Sanger sequencing method, with limited applications (Schmid, Ramos‐Onsins, Ringys‐Beckstein, Weisshaar, & Mitchell‐Olds, 2005; Zhang & Hewitt, 2003). With the advent of next‐generation sequencing (NGS) technologies, the focus of researchers has shifted from single‐genome analysis to multiple‐genome analysis and population studies (Redon et al., 2006). Ever since the first plant genome sequence became available (The Arabidopsis Genome Initiative, 2000), comparative genomic studies have largely focused on single nucleotide polymorphisms (SNPs) in different plant species (Gore et al., 2009; Lai et al., 2015; McNally et al., 2009). A general consensus of the current research is that a single reference genome does not adequately represent the complete genetic content and diversity of a species because of the presence of structural variations (SVs) and TEs, which significantly modify the genetic makeup of different individuals within a species (Saxena, Edwards, & Varshney, 2014). The SVs mainly include presence/absence variants (PAVs) and copy number variants (CNVs) (Supplemental Figure S1). Presence/absence variants are specific sequences or genes that are variably present in individuals of a species and result in extreme structural and phenotypic variations among members of a species (Saxena et al., 2014; Wendel, Jackson, Meyers, & Wing, 2016). Copy number variants are specific sequences or genes present in different copy numbers among members of a species; these varied copy numbers are caused by deletions, insertions, or duplications (Saxena et al., 2014; Scherer et al., 2007). The presence of these SVs in plant genomes and their impact on important agronomic traits of plant species have been widely documented (Supplemental Table S1).

**Core Ideas**

- ncRNAs play important regulatory functions in plant development.
- Recent studies revealed that ncRNAs are evolved from TEs.
- TEs create genomic variation not captured by a single reference genome, affecting ncRNA analyses.
- Pan‐genomes can capture entire variation help to explore functions of ncRNAs.

Several comparative genomic studies in plants indicate that TEs are one of the key factors responsible for extensive variations in both genic and intergenic regions within a species or among closely related species (Morgante, De Paoli, & Radovic, 2007). Brunner, Fengler, Morgante, Tingey, and Rafalski (2005) compared four allelic genomic regions between maize (*Zea mays* L.) inbred lines, B73 and Mo17, and revealed that >50% of the compared sequence differs between these two inbred lines mainly because of TE insertions. Subsequently, Anderson et al. (2019) compared uniformly annotated genome sequences of two more maize genotypes (W22 and PH207) with B73 and Mo17 reference genomes and found that TEs are located within or near 78% of all genes in all four maize genotypes [the authors also identified that 78% of the variable TEs are missing in at least one of the maize genotypes (Anderson et al., 2019)]. Similar genomic diversity has also been observed in other plant species (Golicz, Batley, & Edwards, 2016; Hurgobin & Edwards, 2017; Morgante et al., 2007; Tao, Zhao, Mace, Henry, & Jordan, 2018; Tranchant‐Dubreuil, Rouard, & Sabot, 2019). Additionally, recent studies showed that TEs are the prime source of ncRNAs (Hou et al., 2019). Therefore, characterization of ncRNAs to explore their roles in improving crop productivity, requires better understanding the mobility of TEs and their involvement in genic and nongenic variations. Knowledge of TE underlying genetic variation may provide resources for the identification and functional characterization of ncRNAs.

Analysis of the pan‐genomes is critical for analyzing the complete genomic contents of any given species and for increasing the efficiency of ncRNA discovery. In this review, we focus mainly on the recent progress in plant pan‐genomics, the different approaches available for pan‐genome analysis, and the need for pan‐genomes for ncRNA discovery. We also describe the origin of ncRNAs from TEs and how pan‐genomes could facilitate ncRNA analyses.

## 2 THE PAN‐GENOME PERSPECTIVE

To study phenotypic and genomic variations in a given species and to capture their entire genomic contents, it is vital to construct a pan‐genome (Golicz et al., 2016; Hurgobin & Edwards, 2017; Tahir Ul Qamar, Zhu, Xing, & Chen, 2019; Tao et al., 2018). The concept of a pan‐genome was first introduced in 2005 by Tettelin et al. (2005), who constructed the pan‐genome of a bacterium, *Streptococcus agalactiae*. Subsequently, definitions and objectives of the pan‐genome were modified and interpreted differently by various research groups (Alcaraz et al., 2010; Carlos Guimaraes et al., 2015; Plissonneau, Hartmann, & Croll, 2018; Rasko et al., 2008; Snipen, Almoy, & Ussery, 2009; Tetz, 2005), leading to the notion that a pan‐genome can be either sequence‐based or gene‐based (Golicz, Bayer, Bhalla, Batley, & Edwards, 2020). A sequence‐based pan‐genome refers to a complete collection of nonredundant sequences within members of a species. The advantage of a sequence‐based pan‐genome is that it captures genic as well as nongenic sequences. On the other hand, a gene‐based pan‐genome contains a complete set of genes or orthologous genes families within members of a species (Hubner et al., 2019; Sun et al., 2016; Tranchant‐Dubreuil et al., 2019; Wang et al., 2018).

The pan‐genome is further categorized as the core genome or variable genome (Golicz et al., 2020; Khan et al., 2019). The core genome refers to a common set of sequences or genes present in all individuals of a species and is described as a minimal genome required for an individual to survive and perform basic functions (Gordon et al., 2017; Segerman, 2012; Tranchant‐Dubreuil et al., 2019; Wang et al., 2018). The variable genome (also known as an accessory, dispensable, or shell genome) is a collection of sequences or genes present in only some members of a species (Gordon et al., 2017; Li et al., 2014; Segerman, 2012; Vernikos, Medini, Riley, & Tettelin, 2015) (Supplemental Figure S2a). Genes present in the variable genome of an individual are usually involved in biotic and abiotic stress responses (Tranchant‐Dubreuil et al., 2019). The core and variable genomes are further subdivided into core or soft‐core and variable or member‐specific genomes, respectively (Gordon et al., 2017; Medini, Donati, Tettelin, Masignani, & Rappuoli, 2005; Tranchant‐Dubreuil et al., 2019).

A pan‐genome can also be characterized as open or closed (Khan et al., 2019) (Supplemental Figure S2b). An open pan‐genome contains no fixed number of genes or gene families, and sequencing of additional members of a species gradually increases the size of the pan‐genome. By contrast, in a close pan‐genome, the number of genes or gene families is fixed, and the addition of members does not affect the size of the pan‐genome (Golicz et al., 2016; Hurgobin & Edwards, 2017; Tao et al., 2018; Tranchant‐Dubreuil et al., 2019).

## 3 METHODS FOR PLANT PAN‐GENOME ASSEMBLY AND ANALYSIS

**FIGURE 1:**
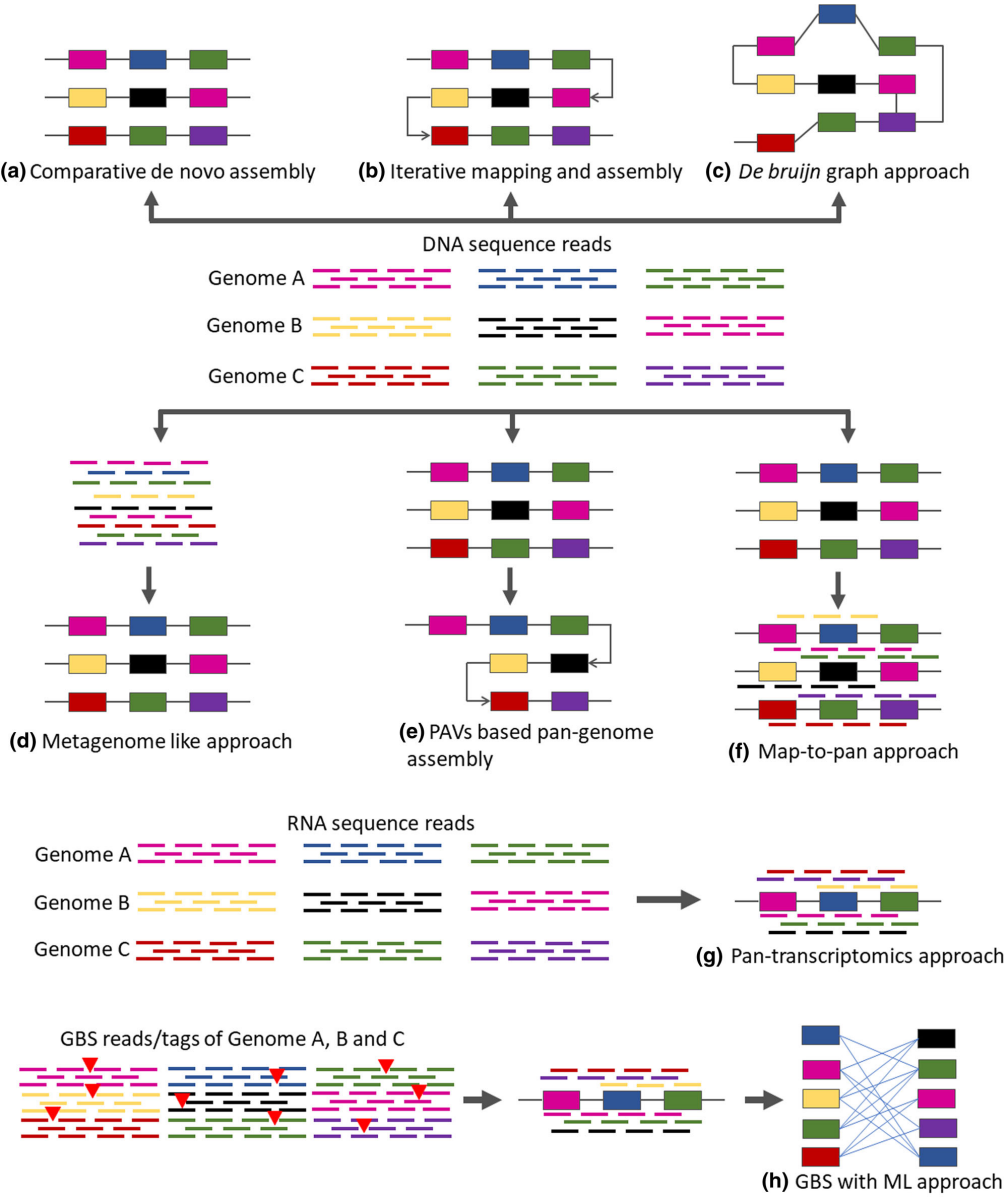
Illustration of different approaches for pan‐genome assembly and analyses. (a) Comparative de novo assembly approach; (b) iterative mapping and assembly approach; (c) de Bruijn graph method; (d) metagenome‐like method; (e) presence/absence variant (PAV)‐based pan‐genome construction method; (f) map‐to‐pan approach; (g) pan‐transcriptomics approach; (h) genotyping‐by‐sequencing (GBS) coupled with machine learning (ML) approach

The method used to develop a pan‐genome, together with the selection of suitable samples, ploidy level of samples, quality of genome assembly and annotation, and the approaches used to detect orthologous genes, greatly influence the quality of pan‐genome analysis (Golicz et al., 2016; Tao et al., 2018). The commonly used methods for constructing plant pan‐genomes (Figure 1) can be categorized into distinct groups described below.

### 3.1 Methods for high‐quality coverage data

If the members of a species have been sequenced with high‐quality coverage, a comparative de novo assembly approach is an excellent choice for pan‐genome analyses. In this method, genomes are separately assembled and annotated, followed by all‐vs.‐all alignment for screening core and variable sequences. This method is costly, time‐consuming, and error‐prone, especially if short‐read sequencing is used for large genomes. However, rapid developments in sequencing technologies, specifically long‐read sequencing technologies, and improvements in assembly tools will gradually reduce the cost of sequencing, minimize the time needed for sequence assembly, and empower the use of the comparative de novo assembly approach for plant pan‐genome analyses in the near future (Golicz et al., 2016; Hurgobin & Edwards, 2017; Tao et al., 2018; Tranchant‐Dubreuil et al., 2019). Additionally, genotyping‐by‐sequencing (GBS) together with the machine‐learning (ML) approach can also be used if members of a species have been sequenced with high‐quality coverage and a high‐quality reference genome is available. First, members are sequenced by GBS, and then the GBS tags are mapped onto a high‐quality reference genome using genome‐wide association study (GWAS) and joint linkage mapping in the nested association mapping population. Furthermore, ML approaches are used to anchor all mapped tags to construct a pan‐genome (Lu et al., 2015).

### 3.2 Methods for low‐quality coverage and short‐read sequencing data

If members of a species have been sequenced at low coverage by short‐read sequencing, iterative mapping and assembly, and map‐to‐pan approaches can be used. In this method, a single whole‐genome assembly is first developed as a pan‐genome reference, which is then used for annotating and mapping all sequence reads. However, iterative mapping and assembly and map‐to‐pan approaches differ in the steps involved in pan‐genome construction. In the iterative mapping and assembly approach, a pan‐genome is constructed by first developing a single whole‐genome assembly reference and then mapping reads from other members of the species serially onto the reference. The reference genome is updated at each turn with assembled unmapped reads and then updated reference genome is used to successively map reads of other members until the final pan‐genome is established. This approach saves time but can cause errors if similar sequences are designated as extra copies, duplicates, novel dispensable sequences, or alleles (Golicz et al., 2016; Hurgobin et al., 2018; Monat et al., 2018; Montenegro et al., 2017; Ou et al., 2018; Pinosio et al., 2016; Tao et al., 2018; Tranchant‐Dubreuil et al., 2019). In the map‐to‐pan approach, a pan‐genome is constructed by de novo assembly of genomes of individual members of a species and then low‐quality assemblies are mapped onto a high‐quality reference genome (Hu et al., 2017; Tao et al., 2018; Wang et al., 2018). Additionally, metagenome‐like and de Bruijn graph methods can also be used for low‐coverage sequencing data. The metagenome‐like method uses a bacterial metagenome‐like approach and assembles all sequences. After assembling whole‐genome sequences of all samples, contigs are reassigned to each member by mapping its data onto the metagenome assembly. This method is compatible with low‐coverage data but may produce errors in the form of chimeric assembly of artificial sequences (Tranchant‐Dubreuil et al., 2019; Yao et al., 2015). In the de Bruijn graph method, all sequences are divided into smaller fragments of *K* length. The association between *K*‐mers of different samples represents graph edges. In the graph, every *K*‐mer represents a node, and overlapping nodes are linked by an edge. A single graph can represent several edges connected by similar nodes that form a network. Information about the origin of a node is crucial if multiple genomes are present. Nodes originating from different genomes can be color coded for tracking purposes, and the whole pan‐genome can be represented as multiple colored graphs. A balance between the total variant number and mapping accuracy is very important since an imbalance between these two parameters may lead to false positive mappings (Golicz et al., 2016; Hurgobin & Edwards, 2017; Iqbal, Caccamo, Turner, Flicek, & McVean, 2012; Lin et al., 2014; Marcus, Lee, & Schatz, 2014). The graph‐based pan‐genome analysis approach is advantageous compared with linear pan‐genome analysis approaches, as it allows storing complex genomic variations in a compact and balanced manner, thus ensuring that their actual annotation remains preserved. Unlike plant pan‐genomics, human pan‐genomics has led to a consensus that graph‐based pan‐genome approaches could overcome the challenge of storing all human sequence variation at a single platform. A comprehensive discussion of graph‐based human pan‐genomics can be found in a recent review by Sherman and Salzberg (2020).

### 3.3 Method for hybridizing different sequencing approaches

In contrast to the method mentioned above, if members of a species have been sequenced by different sequencing approaches, including short reads and long reads, with reasonable coverage and quality, the PAV‐based pan‐genome construction method (Tahir Ul Qamar et al., 2019) would be appropriate for constructing the pan‐genome. This method requires a high‐quality genome assembly as a reference. Then, genomes of other members of the species are iteratively mapped onto the reference genome assembly rather than mapping the reads against the selected reference. Additionally, this method identifies genic and nongenic PAVs and improves and annotates the reference genome successively. This method is less time‐consuming and more cost‐effective.

### 3.4 Method used to construct a pan‐genome using transcriptomic data

An alternative approach for pan‐genome assembly is the pan‐transcriptomics approach, which is mainly based on transcriptomic data, although genomic data can also be used (Hubner et al., 2019). In this method, transcriptomes of all members of a species are sequenced and then all mRNA sequence reads are mapped on to a good quality reference genome. Unmapped reads are de novo assembled, and novel transcripts are identified. Finally, the reference genome is updated to the pan‐genome. This method generates data not only on genomic PAVs but also on transcript PAVs. However, this method does not provide information on nongenic regions (Hirsch et al., 2014; Tao et al., 2018).

### 3.5 Tools available for pan‐genome development and analysis

A few software packages (Danilevicz, Fernandez, Marsh, Bayer, & Edwards, 2020; Xiao, Zhang, Wu, & Yu, 2015) capable of handling small and less complex genomes have been developed for plant pan‐genomics. Most of these software tools, such as Orthofinder (Emms & Kelly, 2015), OrthoMCL (Li, Stoeckert, & Roos, 2003), and Get_Homologues‐EST (Contreras‐Moreira et al., 2017), can annotate core and variable genes. However, some of these tools, including EUPAN (Hu et al., 2017) and ppsPCP (Tahir Ul Qamar et al., 2019), can construct sequence‐based pan‐genomes (Table 1).

**TABLE 1:** Tools available for plant pan‐genome analyses

Software	Description	Reference
Orthofinder	A fast and accurate tool that finds orthologs and orthogroups, infers gene trees for each orthogroup, and infers a rooted species tree for each species being analyzed. It takes protein sequences as input, and can be further used to analyze core and variable genes. Availability: https://github.com/davidemms/OrthoFinder	Emms & Kelly, 2015
OrthoMCL	A pipeline that clusters orthologous protein sequences based on their sequence similarity, and offers information about core and variable portions of the pan‐genome. It uses amino acid sequences as the input. Furthermore, it groups representative species‐specific gene expansion families and improves genome annotation. Availability: https://orthomcl.org/orthomcl/	Li et al., 2003
Get_Homologues‐EST	A very comprehensive pipeline that clusters orthologous genes, analyzes core and variable genomes, and generates core/variable genome curves. It uses coding sequences (CDSs) as the input. Amino acid sequences of the respective CDSs can also be supplied as the input. Availability: https://github.com/eead-csic-compbio/get_homologues	Contreras‐Moreira et al., 2017
EUPAN	An inclusive toolkit for plant pan‐genomes that follows the “map‐to‐pan” approach and assembles large‐scale pan‐genomes. It uses raw reads as input. Availability: http://cgm.sjtu.edu.cn/eupan/	Hu et al., 2017
ppsPCP	A unique, fast, and accurate pipeline, which takes advantage of the previously assembled genomes for scanning PAVs, and generates a fully‐annotated pan‐genome of eukaryotes as well as prokaryotes. This pipeline is more accessible than other pipelines for non‐bioinformaticians. Availability: https://github.com/Zhuxitong/ppsPCP	Tahir Ul Qamar et al., 2019

## 4 ADVANCES IN PLANT PAN‐GENOME STUDIES

With advancements in high‐throughput sequencing technologies and rapid reduction in the cost of genome sequencing, several pan‐genome studies have recently been initiated to evaluate the diversity and evolution of plant species to gain a better understanding of the core and variable genes of crop plants and to identify missing candidate genes underlying phenotypic variations (Golicz et al., 2016; Hurgobin & Edwards, 2017; Tao et al., 2018). Pan‐genomes have been constructed for many plant species including *Arabidopsis thaliana* (L.) Heynh. (Contreras‐Moreira et al., 2017), maize (Hirsch et al., 2014; Lu et al., 2015), soybean [*Glycine max* (L.) Merr.] (Li et al., 2014), *Brassica* spp. (Golicz et al., 2016; Hurgobin et al., 2018; Lin et al., 2014), rice (*Oryza sativa* L.) (Schatz et al., 2014; Wang et al., 2018; Yao et al., 2015; Zhao et al., 2018), wheat (*Triticum aestivum* L.) (Montenegro et al., 2017), *Medicago truncatula* Gaertn. (Zhou et al., 2017), *Brachypodium distachyon* (L.) P. Beauv. (Gordon et al., 2017), poplar (*Populus* spp.) (Pinosio et al., 2016), pepper (*Capsicum annuum* L.) (Ou et al., 2018), sesame (*Sesamum indicum* L.) (Yu et al., 2019), sunflower (*Helianthus annuus* L.) (Hubner et al., 2019), tomato (*Solanum lycopersicum* L.) (Gao et al., 2019), and pigeon pea (*Cajanus cajan* L. Huth) (Zhao et al., 2020) (Table 2).

**TABLE 2:** Overview of pan‐genome studies in various plant species

Method	Species	Sample size	Total no. of pan‐genes	Core gene	Variable genes	Reference
				%		
Comparative de novo assembly	*Arabidopsis thaliana*	19	37,789	69.7	30.3	Contreras‐Moreira et al., 2017
	*Oryza sativa*	3	40,362	92.17	7.83	Schatz et al., 2014
	*O. sativa*, *O. rufipogon*	66	42,580	61.94	38.06	Zhao et al., 2018
	*Glycine soja*	7	59,080	48.60	51.40	Li et al., 2014
	*Brachypodium distachyon*	54	37,886	55.00	45.00	Gordon et al., 2017
	*Medicago truncatula*	15	74,700	41.9	58.1	Zhou et al., 2017
	*Lycopersicum esculentum*	725	40,283	74.2	25.8	Gao et al., 2019
Iterative mapping and assembly	*Brassica oleracea*	9	61,379	81.29	18.71	Golicz et al., 2016
	*Brassica napus*	53	94,013	62.26	37.74	Hurgobin et al., 2018
	*Triticum aestivum*	18	140,500	57.70	42.30	Montenegro et al., 2017
	*Capsicum annuum*	355	51,757	55.7	44.3	Ou et al., 2018
	*O. glaberrima*	120 cultivars/74 wild accessions	35,198/36,252	86.5/98.6	13.5/1.4	Monat et al., 2018
	*Cajanus cajan*	89	55,512	86.6	13.4	Zhao et al., 2020
Pan‐transcriptomics	*Zea mays*	503	41,903	39.12	60.88	Hirsch et al., 2014
	*Helianthus annuus*	493	61,205	72.7	27.3	Hubner et al., 2019
GBS with ML	*Z. mays*	14,129	–	–	–	Lu et al., 2015
de Bruijn graph	*Brassica rapa*	3	36,882	84.56	15.44	Lin et al., 2014
Metagenome‐ like	*O. sativa*	1,483	–	–	–	Yao et al., 2015
Map‐to‐pan	*O. sativa*	3,010	48,098	48.5‐58.3	41.7‐51.5	Wang et al., 2018
Mapping only	*Populus* spp.	22	–	80.7	19.3	Pinosio et al., 2016
	*Sesamum indicum*	4	–	58.21	41.79	Yu et al., 2019
PAV‐based	*Brassica* *Napus*	8	152,185	56.00	44.00	Song et al., 2020

Plant pan‐genomes are mainly based on the knowledge of TEs and SVs (Golicz et al., 2016; Morgante et al., 2007; Tao et al., 2018). The core genome in plant pan‐genomes is composed of conserved genes that have lower SNP density and longer average length and are usually involved in basic cellular functions. By contrast, the dispensable genome in plant pan‐genomes is based on genes that enable plants to withstand environmental stresses, have higher SNP density and nonsynonymous to synonymous substitution ratio (Ka/Ks), have shorter average length, and are likely involved in structural and functional diversity (Golicz et al., 2016; Tao et al., 2018). Although the available pan‐genome data are not readily comparable, since they are based on different sample sizes and assembly approaches, the dispensable genome in plant pan‐genomes varies from 8–61% (regardless of the method used for analysis), and the available methods of pan‐genome analyses are unable to capture all functional variation to fully explore the dispensable genes (Tao et al., 2018).

In maize, two pan‐genome studies have been reported. Hirsch et al. (2014) developed a closed pan‐genome of 503 maize inbred lines using the pan‐transcriptomics approach. The authors revealed that 8,681 representative transcript assemblies were missing in the B73 maize reference genome (Hirsch et al., 2014). In another study of 14,129 maize inbred lines, Lu et al. (2015) developed a pan‐genome using GBS and ML and revealed 1.1 million PAV tags.

In rice, four pan‐genome studies have been published to date. Schatz et al. (2014) studied three divergent rice species using a comparative de novo assembly approach and revealed that 92% of all genes are core genes and only the remaining ∼8% are variable genes. The authors also showed that variable genes in rice have a lower number of exons than core genes (Schatz et al., 2014). Yao et al. (2015) explored the dispensable genome of 1,483 rice cultivators using the metagenome‐like method and showed that 8,000 genes are absent from the Nipponbare rice reference genome. Zhao et al. (2018) constructed a pan‐genome of 66 rice species (57 divergent accessions and nine modern cultivars) using the comparative de novo assembly approach and identified 23 million intergenomic sequence variants. In another study, Wang et al. (2018) established a closed pan‐genome of rice based on 3,010 diverse Asian cultivars and found more than 90,000 SVs and over 10,000 novel full‐length protein‐coding genes. The study involving 3,010 rice cultivars showed a higher percentage of variable genome (∼41%) compared with the study involving only three rice cultivars (8% variable genome). This clearly shows that the sample size influences pan‐genome studies; however, increase in sample size can lead to a point where any further increase would not lead to a further expansion of the pan‐genome size. In addition, the study of (Tao et al., 2018) involving 66 diverse species of rice revealed 42,580 genes (38% variable) compared with the study of (Wang et al., 2018) involving 3,010 Asian cultivars, which revealed 48,098 genes (41% variable). This clearly demonstrates that the type of samples used in the study (i.e., similar or diverse) also influences pan‐genome analyses.

The pan‐genome of *Brassica* species has been investigated in four studies. Lin et al. (2014) compared and functionally annotated three *B. rapa* genomes {turnip (*B. rapa* subsp. *rapa*), rapid‐cycling *B. rapa*, and Chinese cabbage [*B. rapa* subsp. *Pekinensis* (Lour.) Hanelt]} using de Bruijn graph method and revealed significant divergence prior to their domestication. Golicz et al. (2016) studied the closed pan‐genome of cabbage (*Brassica oleracea* L.) based on nine cultivars using the iterative mapping and assembly approach and found that ∼80% of all genes are core genes, while the remaining ∼20% are affected by PAVs. Hurgobin et al. (2018) constructed the pan‐genome of rape (*Brassica napus* L.) based on 53 cultivars using the iterative mapping and assembly approach and showed that 62% of pan‐genes are core genes and 38% of genes are variable possibly because of homologous exchange. In a recent study, Song et al. (2020) constructed the pan‐genome of rape based on eight cultivars using the PAV‐based approach. The authors showed that 56% of pan‐genes are core genes, 42% of genes are variable, and the remaining 2% are species‐specific (Song et al., 2020). Details of other plant pan‐genomes are listed in Table 2.

## 5 PAN‐GENOMES AND TRANSPOSABLE ELEMENTS

Transposable elements are defined as genomic elements capable of moving from one location to another in the genome and represent one of the major components of eukaryotic genomes especially plants. Transposable elements are categorized into two classes: Class I (retrotransposons) and Class II (DNA transposons) (Cho, 2018; Feschotte, Jiang, & Wessler, 2002; Hadjiargyrou & Delihas, 2013; Morgante et al., 2007; Wicker et al., 2007). Retrotransposons move from one location to another via RNA intermediates, which are later converted into cDNAs, thus creating several extra copies in the genome. Retrotransposons mainly include long terminal repeat (LTR) retrotransposons, long interspersed nuclear elements, and short interspersed nuclear elements (Cho, 2018). Long terminal repeat retrotransposons are predominant in plant genomes, and their amplification is believed to be one of the key factors responsible for a substantial increase in genome size. Several studies indicate that amplification of certain LTR retrotransposon families can cause significant variation in genome size among closely related species (Bennetzen, 2002), and certain LTR retrotransposons can also induce gene translocation and chromosomal rearrangement (Xiao, Jiang, Schaffner, Stockinger, & van der Knaap, 2008). Hawkins, Kim, Nason, Wing, and Wendel (2006) showed that amplification of *Gorge3* LTR retrotransposon families led to a substantial increase in the genome size of cotton (*Gossypium* spp.). The latest studies suggest that LTR retrotransposons jump into the enhancer, repressor, and promoter regions of genes to regulate their expression (Kashkush, Feldman, & Levy, 2002; Schramke & Allshire, 2003).

DNA transposons move in the genome using a copy‐and‐paste mechanism, which involves rolling circle amplification. DNA transposons are further divided into two subclasses: Helitrons and miniature inverted‐repeat transposable elements (MITEs) (Cho, 2018; Liu et al., 2019; Morgante et al., 2007). These MITEs regulate the expression of host genes and are usually found in close proximity to genic regions (Piriyapongsa, Marino‐Ramirez, & Jordan, 2007; Zerjal, Joets, Alix, Grandbastien, & Tenaillon, 2009). The insertion and removal of MITEs can lead to PAVs in the host genome, which is thought to be an important aspect of host genome evolution and diversity (Sampath et al., 2014; Sun et al., 2020).

**FIGURE 2:**
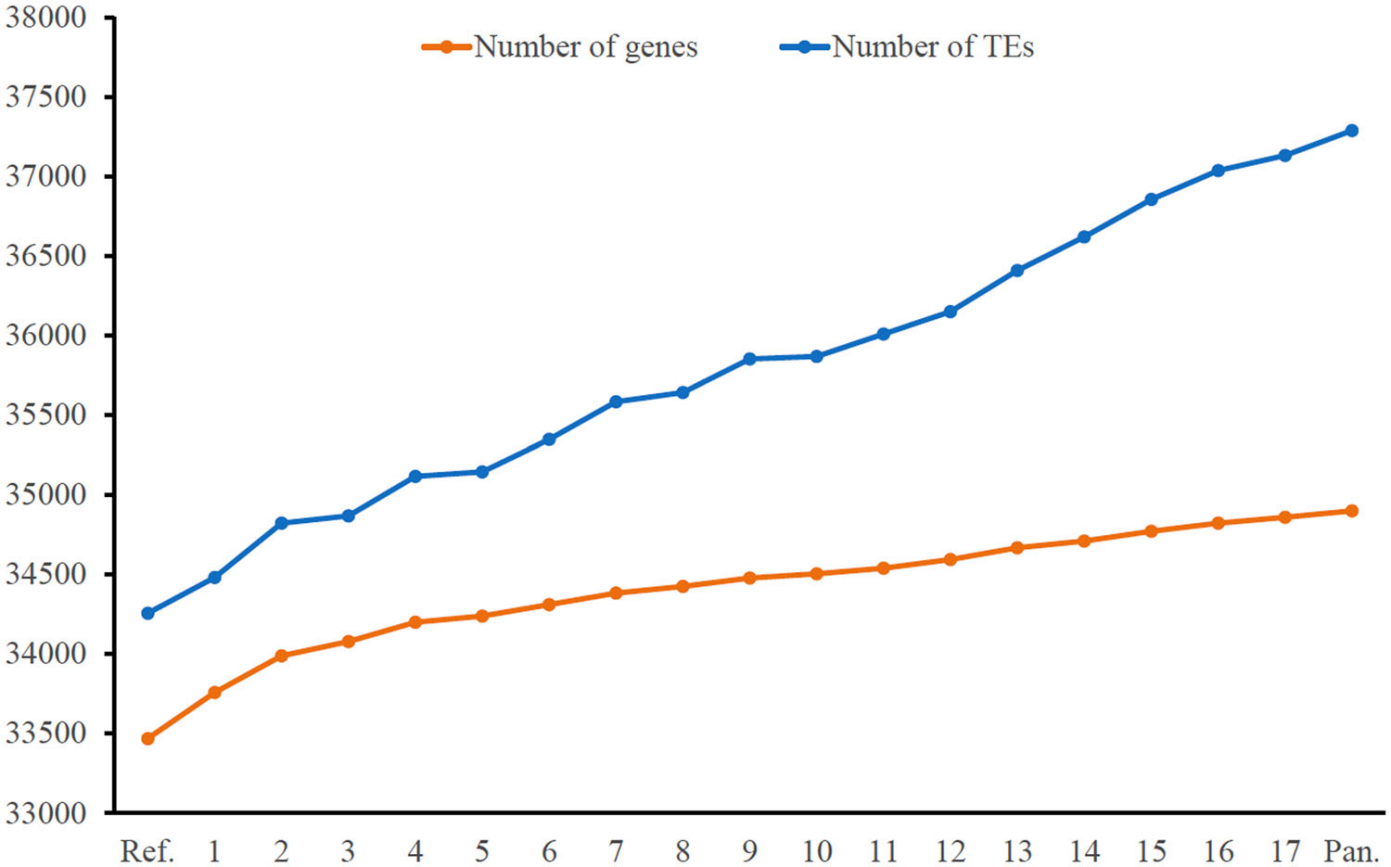
Number of pan‐genes and pan‐transposable elements (TEs) contributed by each ecotype of *A. thaliana* to the gradually developing pan‐genome. Ref, reference genome (Col‐0); 1–17, query genomes (Bur‐0, Can‐0, Ct‐1, Edi‐0, Hi‐0, Kn‐0, Ler‐0, Mt‐0, No‐0, Oy‐0, Po‐0, Rsch‐4, Sf‐2, Tsu‐0, Wil‐2, Ws‐0, Wu‐0, and Zu‐0, respectively; Pan, pan‐genome

Recent high‐throughput sequencing data show that plant genomes are highly dynamic, plastic, and variable, and TEs are largely responsible for the SV in plant genomes (Cho, 2018; Liu et al., 2019; Morgante et al., 2007). Transposable elements are widely documented in several important crops including maize, rice, wheat, and barley (*Hordeum vulgare* L.) and constitute at least 50% of the genomes of cereal crops (Cho, 2018; Hadjiargyrou & Delihas, 2013; Tenaillon, Hollister, & Gaut, 2010). Recently, we developed a pan‐genome of model plant Arabidopsis using whole‐genome sequence assemblies of 19 ecotypes (Tahir Ul Qamar et al., 2019) (http://cbi.hzau.edu.cn/ppsPCP/) and identified TEs in the gradually developing pan‐genome using RepeatMasker (Tarailo‐Graovac & Chen, 2009) with default parameters. Our results showed that as the number of pan‐genes increased with the addition of each assembly to the developing pan‐genome the number of pan‐TEs also increased (Supplemental Table S2). Interestingly, the curve of pan‐TEs was more open than that of pan‐genes (Figure 2), possibly because of the less conserved and more variable nature of TEs. The reference genome of *A. thaliana* ecotype Columbia (Col‐0) contains 33,467 genes and 34,255 TEs, whereas Arabidopsis ecotypes used to establish the pan‐genome presented 34,899 pan‐genes and 37,288 pan‐TEs. In total, 3,033 new TEs were identified in the Arabidopsis pan‐genome, which were absent in the Col‐0 reference genome. These results further confirm that pan‐genomes are crucial for characterizing the diversity of a species and for exploring and exploiting the mobility and functions of TEs and their involvement in genic and nongenic variations.

## 6 TRANSPOSABLE ELEMENTS AND NONCODING RNA

**FIGURE 3:**
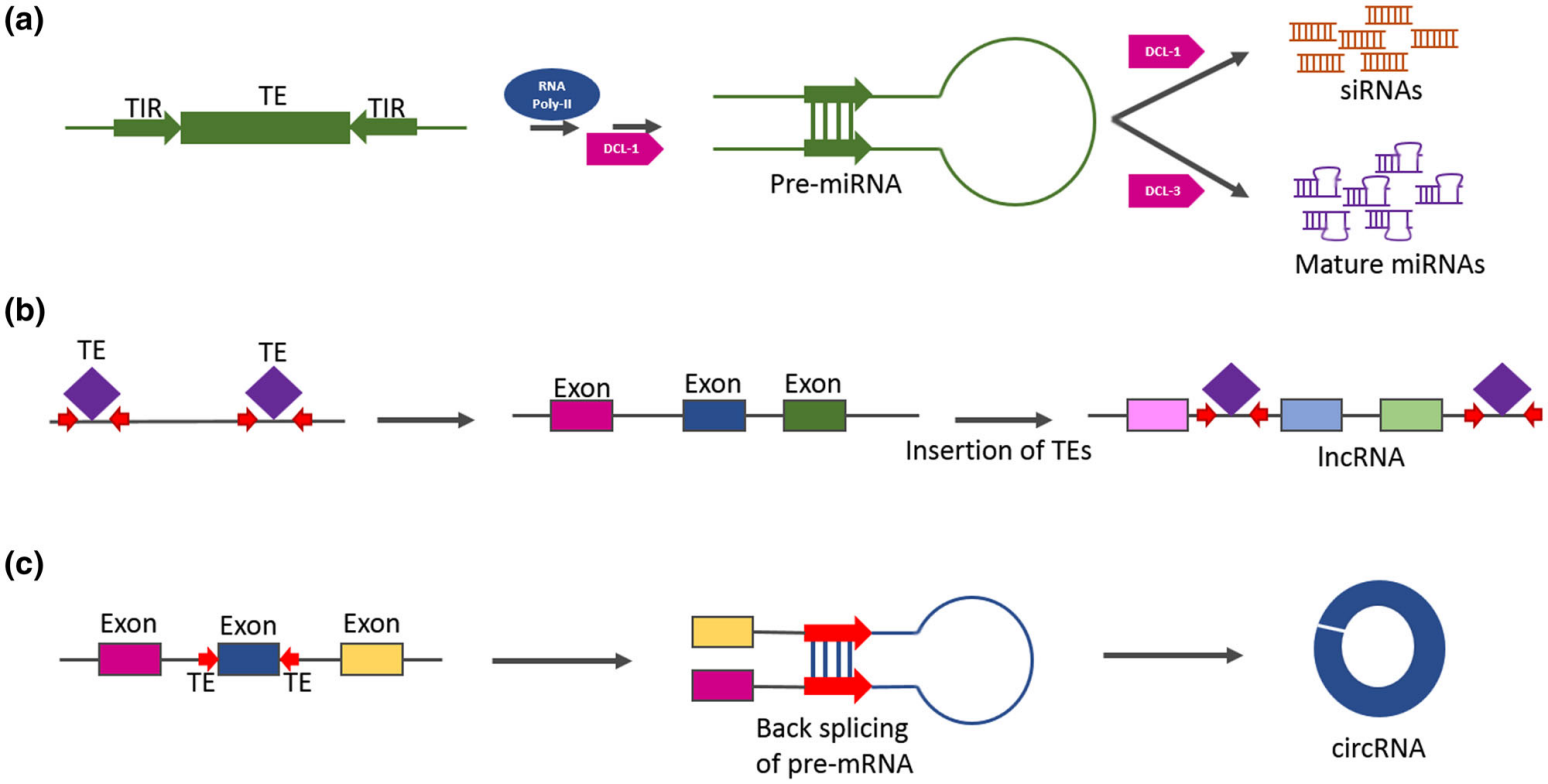
Origin of noncoding RNAs (ncRNAs) from transposable elements (TEs). (a) Schematic representations showing the possible mechanisms of the origin of small interfering RNAs (siRNAs) and microRNAs (miRNAs) from TEs, (b) long ncRNAs (lncRNAs) from TEs, and (c) circular RNA (circRNA) from TEs via back‐splicing

The mobility of TEs in a genome can cause mutagenesis. Thus, host genomes have evolved special epigenetic silencing pathways involving DNA methylation, RNA‐directed DNA methylation, and histone modifications to suppress TE activity and ensure genome stability (Baulcombe, 2004; Blevins et al., 2015; Cho, 2018; Fultz, Choudury, & Slotkin, 2015; Li et al., 2015; Matzke & Mosher, 2014; Zhai et al., 2015). Studies suggest, in the result of suppression, TEs mediated non–ncRNAs have been produced (Figure 3a) (Hou et al., 2019). For example, few MITE transcripts can form hairpin‐like structures, which are recognized by dicer‐like enzymes and cleaved into small RNAs, especially siRNAs. This phenomenon has been confirmed in Arabidopsis, rice, and humans. The MITE‐derived small RNAs account for 25% of all small RNAs in the rice genome, which clearly shows that TEs play critical roles in the regulation of plant biological processes (Liu et al., 2019; Lu et al., 2011; Piriyapongsa & Jordan, 2008; Piriyapongsa et al., 2007). Some TEs are methylated via DNA methylation pathways, transcribed by plant‐specific RNA polymerase IV (Pol IV), duplexed by RNA‐dependent RNA polymerase (RdRP), and further cleaved by dicer‐like enzymes to form siRNAs (Blevins et al., 2015; Cho, 2018; Li et al., 2015; Zhai et al., 2015; Zhong et al., 2014). Recent studies indicate that like siRNAs, some miRNAs also originate from TEs (Li et al., 2011; Lorenzetti, de Antonio, Paschoal, & Domingues, 2016; Piriyapongsa & Jordan, 2008). Lorenzetti et al. (2016) identified 152 miRNAs overlapping with TEs in 10 different plant species. Multiple studies showed that hairpin‐like structures formed by TEs can be further recognized by miRNA biogenesis pathways and cleaved into miRNAs (Cho, 2018; Li et al., 2011; Piriyapongsa & Jordan, 2008).

Moreover, recent studies show that a large number of lncRNAs and circRNAs originate from TE transcripts (Figure 3b,c), thus reinforcing the dynamic evolutionary role of TEs in ncRNA biogenesis (Chen et al., 2018; Kapusta et al., 2013; Kelley & Rinn, 2012; Liang & Wilusz, 2014; Liu et al., 2012; Z. P. Wang et al., 2017). Recently, D. Wang et al. (2017) studied lncRNAs in Arabidopsis, rice, and maize under different abiotic stress conditions and identified 47,611 and 398 TE‐associated lncRNAs, respectively (Cho, 2018; D. Wang et al., 2017). In maize, Z. P. Wang et al. (2017) showed that sequences related to LINE1‐like elements and their reverse complementary pairs (LLERCPs) are significantly enriched in regions flanking circRNAs. The authors concluded that TEs could potentially be involved in the development of circRNAs in plants (Z. P. Wang et al., 2017). Chen et al. (2018) also studied the association of TEs with circRNAs in maize and found similar results. Genes overlapping LLERCP‐derived circRNAs are mostly enriched in loci associated with phenotypic variation. These results strengthen the concept that circRNAs likely originate from TEs and play important roles in phenotypic variation (Chen et al., 2018).

## 7 HOW PLANT PAN‐GENOMES CAN INCREASE THE EFFICIENCY OF NONCODING RNA DISCOVERY

The origin of regulatory ncRNAs is not well understood. Besides the role of TEs and other mechanisms of ncRNA biogenesis have been proposed recently (Hou et al., 2019). For example, miRNAs are hypothesized to originate (a) from inverted duplication of pre‐existing protein‐coding genes (Tanzer & Stadler, 2004; Zhang, Jiang, & Gao, 2011) and (b) through endogenous hairpins that create stem‐loop structures in intronic or intergenic regions (Brodersen et al., 2008; Hou et al., 2019). Similarly, lncRNAs are also thought to originate from the duplication of pre‐existing lncRNAs and by the decay of pseudo‐protein‐coding genes (Hou et al., 2019). Additionally, circRNAs can originate from exons, introns, or intergenic regions of protein‐coding genes (Hou et al., 2019). Although the presence of these ncRNAs (miRNAs, lncRNAs, and circRNAs) in major crops has been documented recently (Table 3), their mechanisms of biogenesis and functions remain poorly understood.

**TABLE 3:** Number of predicted microRNAs (miRNAs), long noncoding RNAs (lncRNAs), and circular RNAs (circRNAs) in plant species

Plant species	No. of miRNAs	No. of lncRNAs	No. of circRNAs	References
*Arabidopsis thaliana*	1,581	3,008	38,938	PlantcircBase, GREENC and PNRD databases (Chu et al., 2017; Paytuví Gallart, Hermoso Pulido, Anzar Martínez de Lagrán, Sanseverino, & Aiese Cigliano, 2015; Yi, Zhang, Ling, Xu, & Su, 2014)
*Oryza sativa*	2,819	5,237	40,311	
*Zea mays*	325	18,110	6,302	
*Triticum aestivum*	384	38,820	129	
*Gossypium*	717	4,216	3,652	
*Glycine max*	664	6,689	7,806	
*Solanum tuberosum*	364	6,680	1,728	

**FIGURE 4:**
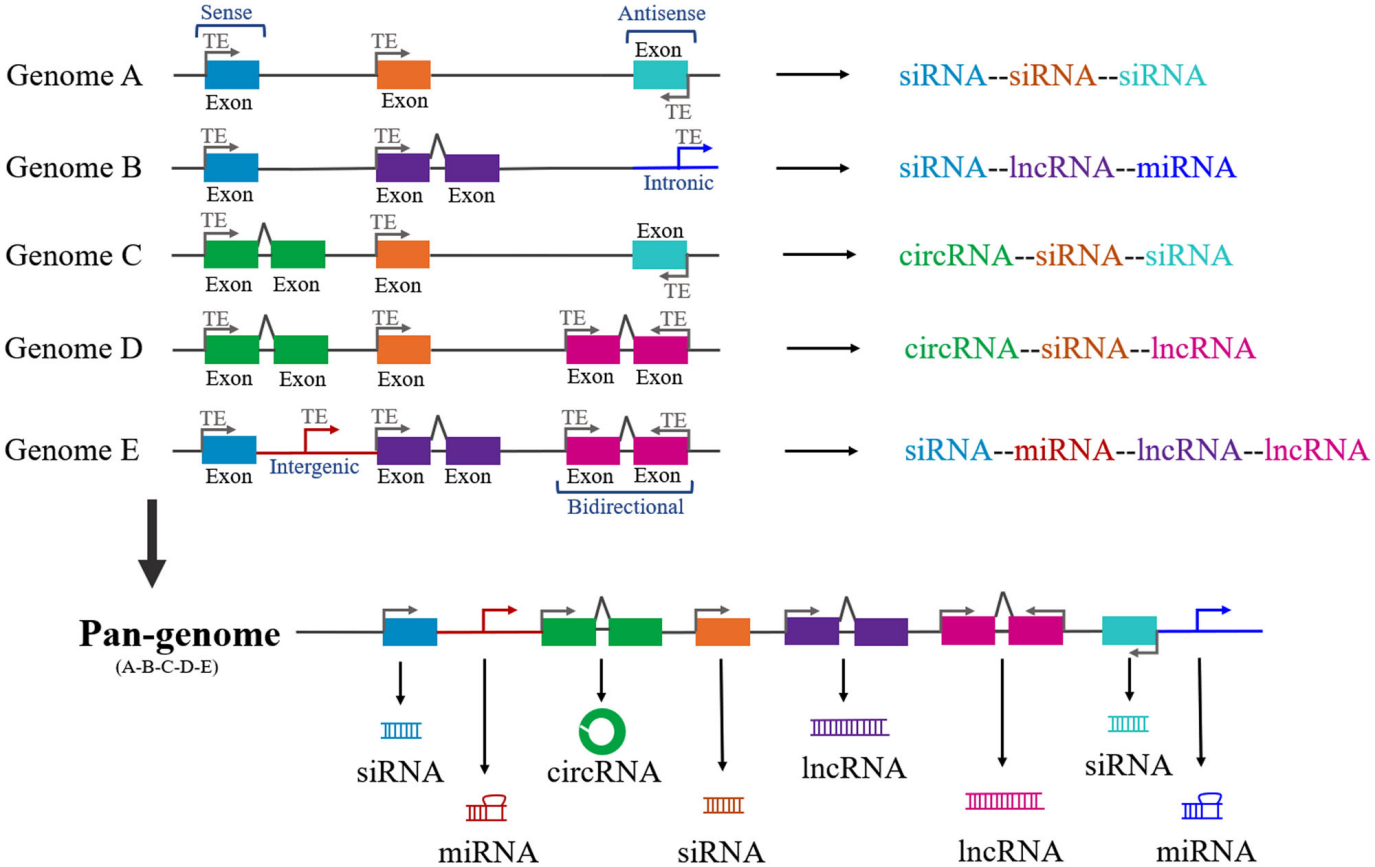
Schematic representation of the importance of pan‐genomes in noncoding RNA (ncRNA) discovery and analysis

Recent studies suggest that improvements are required in ncRNA detection procedures. Using a pan‐genome for predicting ncRNAs will increase the efficiency of predictions and the overall number of ncRNAs predicted compared with using a single reference genome. Because a pan‐genome is a collection of all coding and noncoding sequences of all members of a species, it presents a more accurate and complete set of genomic contents (Figure 4). For example, in a recent study, Gao et al., (2019) constructed a pan‐genome for tomato and revealed that 4,873 crucial genes involved in important agronomic traits were missing in the tomato reference genome. Moreover, if a pan‐genome is used for ncRNA discovery, reads from several members of a species can be simultaneously mapped to the pan‐genome, and there will be no need for matching or merging the results generated using different reference genomes to consolidate a single ncRNA data set. Thus, using a pan‐genome for ncRNA discovery will significantly save labor, time, and computational resources. This approach will also enable further investigation of a variable genome and will help determine the exact number, type, and function of ncRNAs contributed by each individual to the core and variable portions of the pan‐genome.

## 8 CONCLUSIONS

The biological mechanisms and functions of ncRNAs, previously overlooked, have recently been explored. The discovery of ncRNAs has largely been influenced by the repetitive nature of TEs, short‐read length of NGS data, and inability of a single reference genome to represent the entire diversity of a species. For example, short reads generated by NGS cause ambiguity and missannotation while mapping ncRNAs reads. A single genome cannot represent the complete set of ncRNAs of a species. Therefore, if reads do not align accurately or cannot find target sites in the reference genome, the number of ncRNAs is over‐ or underestimated, thus affecting downstream analyses. It is believed that analyses using a pan‐genome, rather than a single reference genome, will capture the entire genomic contents and genetic diversity of a species. Thus, we conclude that the unknown functions of ncRNAs in plants can be explored using the pan‐genome as a reference.

## AUTHOR CONTRIBUTIONS

L.L.C. and M.T.Q conceived and designed this study. M.T.Q. performed literature review and wrote the paper. L.L.C., X.Z., M.S.K. and F.X. analyzed the data and contribute in paper improvements.

## COMPETING INTERESTS

The authors declare no competing interests.

## Supporting information

Supplemental Figure S1. Illustration of the major types of sequence variations in plants responsible for their genomic and phenotypic diversity.
Supplemental Figure S2. General representation of different portions and types of pan‐genomes.
Supplemental Table S1. Examples of presence/absence variations (PAVs) and copy number variations (CNVs) and their impact on important agronomic traits of major crops (table modified from Tao, Zhao, Mace, Henry, and Jordan [2018])
Supplemental Table S2. Number of genes and transposable elements (TEs) contributed by each Arabidopsis thaliana ecotype to the pan‐genome.

## References

[tpg220046-bib-0001] Alcaraz, L. D. , Moreno‐Hagelsieb, G. , Eguiarte, L. E. , Souza, V. , Herrera‐Estrella, L. , & Olmedo, G. (2010). Understanding the evolutionary relationships and major traits of *Bacillus* through comparative genomics. BMC Genomics, 11, 332. 10.1186/1471-2164-11-332 20504335 PMC2890564

[tpg220046-bib-0002] Anderson, S. N. , Stitzer, M. C. , Brohammer, A. B. , Zhou, P. , Noshay, J. M. , O'Connor, C. H. , … Springer, N. M. (2019). Transposable elements contribute to dynamic genome content in maize. The Plant Journal, 100, 1052–1065. 10.1111/tpj.14489 31381222

[tpg220046-bib-0003] The Arabidopsis Genome Initiative . (2000). Analysis of the genome sequence of the flowering plant *Arabidopsis thaliana* . Nature, 408, 796. 10.1038/35048692 11130711

[tpg220046-bib-0004] Bartel, D. P. (2004). MicroRNAs: Genomics, biogenesis, mechanism, and function. Cell, 116, 281–297. 10.1016/s0092-8674(04)00045-5 14744438

[tpg220046-bib-0005] Baulcombe, D. (2004). RNA silencing in plants. Nature, 431, 356–363. 10.1038/nature02874 15372043

[tpg220046-bib-0006] Bennetzen, J. L. (2002). Mechanisms and rates of genome expansion and contraction in flowering plants. Genetica, 115, 29–36. 10.1023/a:1016015913350 12188046

[tpg220046-bib-0007] Blevins, T. , Podicheti, R. , Mishra, V. , Marasco, M. , Wang, J. , Rusch, D. , … Pikaard, C. S. (2015). Identification of Pol IV and RDR2‐dependent precursors of 24 nt siRNAs guiding de novo DNA methylation in Arabidopsis. Elife, 4, e09591. 10.7554/eLife.09591 26430765 PMC4716838

[tpg220046-bib-0008] Borges, F. , Parent, J. S. , van Ex, F. , Wolff, P. , Martinez, G. , Kohler, C. , & Martienssen, R. A. (2018). Transposon‐derived small RNAs triggered by miR845 mediate genome dosage response in Arabidopsis. Nature Genetics, 50, 186–192. 10.1038/s41588-017-0032-5 29335544 PMC5805582

[tpg220046-bib-0009] Brodersen, P. , Sakvarelidze‐Achard, L. , Bruun‐Rasmussen, M. , Dunoyer, P. , Yamamoto, Y. Y. , Sieburth, L. , & Voinnet, O. (2008). Widespread translational inhibition by plant miRNAs and siRNAs. Science, 320, 1185–1190. 10.1126/science.1159151 18483398

[tpg220046-bib-0010] Brunner, S. , Fengler, K. , Morgante, M. , Tingey, S. , & Rafalski, A. (2005). Evolution of DNA sequence nonhomologies among maize inbreds. Plant Cell, 17, 343–360. 10.1105/tpc.104.025627 15659640 PMC548811

[tpg220046-bib-0011] Carlos Guimaraes, L. , Benevides de Jesus, L. , Vinicius Canario Viana, M. , Silva, A. , Thiago Juca Ramos, R. , de Castro Soares, S. , & Azevedo, V. (2015). Inside the pan‐genome—Methods and software overview. Current Genomics, 16, 245–252. 10.2174/1389202916666150423002311 27006628 PMC4765519

[tpg220046-bib-0012] Chen, L. , Zhang, P. , Fan, Y. , Lu, Q. , Li, Q. , Yan, J. , … Li, L. (2018). Circular RNAs mediated by transposons are associated with transcriptomic and phenotypic variation in maize. New Phytologists, 217, 1292–1306. 10.1111/nph.14901 29155438

[tpg220046-bib-0013] Cho, J. (2018). Transposon‐derived non‐coding RNAs and their function in plants. Frontiers in Plant Science, 9, 600. 10.3389/fpls.2018.00600 PMC5943564 29774045

[tpg220046-bib-0014] Chu, Q. , Zhang, X. , Zhu, X. , Liu, C. , Mao, L. , Ye, C. , … Fan, L. (2017). PlantcircBase: A database for plant circular RNAs. Molecular Plant, 10, 1126–1128. 10.1016/j.molp.2017.03.003 28315753

[tpg220046-bib-0015] Contreras‐Moreira, B. , Cantalapiedra, C. P. , Garcia‐Pereira, M. J. , Gordon, S. P. , Vogel, J. P. , Igartua, E. , … Vinuesa, P. (2017). Analysis of plant pan‐genomes and transcriptomes with GET_HOMOLOGUES‐EST, a clustering solution for sequences of the same species. Frontiers in Plant Science, 8, 184. 10.3389/fpls.2017.00184 28261241 PMC5306281

[tpg220046-bib-0016] Danilevicz, M. F. , Fernandez, C. G. T. , Marsh, J. I. , Bayer, P. E. , & Edwards, D. (2020). Plant pangenomics: Approaches, applications and advancements. Current Opinion in Plant Biology, 54, 18–25. 10.1016/j.pbi.2019.12.005 31982844

[tpg220046-bib-0017] Emms, D. M. , & Kelly, S. (2015). OrthoFinder: Solving fundamental biases in whole genome comparisons dramatically improves orthogroup inference accuracy. Genome Biology, 16, 157. 10.1186/s13059-015-0721-2 26243257 PMC4531804

[tpg220046-bib-0018] Fahlgren, N. , Howell, M. D. , Kasschau, K. D. , Chapman, E. J. , Sullivan, C. M. , Cumbie, J. S. , … Carrington, J. C. (2007). High‐throughput sequencing of *Arabidopsis* microRNAs: Evidence for frequent birth and death of *MIRNA* genes. PLoS ONE, 2, e219. 10.1371/journal.pone.0000219 17299599 PMC1790633

[tpg220046-bib-0019] Feschotte, C. , Jiang, N. , & Wessler, S. R. (2002). Plant transposable elements: Where genetics meets genomics. Nature Reviews Genetics, 3, 329–341. 10.1038/nrg793 11988759

[tpg220046-bib-0020] Fultz, D. , Choudury, S. G. , & Slotkin, R. K. (2015). Silencing of active transposable elements in plants. Current Opinion in Plant Biology, 27, 67–76. 10.1016/j.pbi.2015.05.027 26164237

[tpg220046-bib-0021] Gao, L. , Gonda, I. , Sun, H. , Ma, Q. , Bao, K. , Tieman, D. M. , … Fei, Z. (2019). The tomato pan‐genome uncovers new genes and a rare allele regulating fruit flavor. Nature Genetics, 51, 1044–1051. 10.1038/s41588-019-0410-2 31086351

[tpg220046-bib-0022] Golicz, A. A. , Batley, J. , & Edwards, D. (2016). Towards plant pangenomics. Plant Biotechnology Journal, 14, 1099–1105. 10.1111/pbi.12499 26593040 PMC11388911

[tpg220046-bib-0023] Golicz, A. A. , Bayer, P. E. , Barker, G. C. , Edger, P. P. , Kim, H. , Martinez, P. A. , … Edwards, D. (2016). The pangenome of an agronomically important crop plant *Brassica oleracea* . Nature Communications, 7, 13390. 10.1038/ncomms13390 PMC5114598 27834372

[tpg220046-bib-0024] Golicz, A. A. , Bayer, P. E. , Bhalla, P. L. , Batley, J. , & Edwards, D. (2020). Pangenomics comes of age: From bacteria to plant and animal applications. Trends in Genetics, 36, 132–145. 10.1016/j.tig.2019.11.006 31882191

[tpg220046-bib-0025] Gordon, S. P. , Contreras‐Moreira, B. , Woods, D. P. , Des Marais, D. L. , Burgess, D. , Shu, S. , … Vogel, J. P. (2017). Extensive gene content variation in the *Brachypodium distachyon* pan‐genome correlates with population structure. Nature Communications, 8, 2184. 10.1038/s41467-017-02292-8 PMC5736591 29259172

[tpg220046-bib-0026] Gore, M. A. , Chia, J. M. , Elshire, R. J. , Sun, Q. , Ersoz, E. S. , Hurwitz, B. L. , … Buckler, E. S. (2009). A first‐generation haplotype map of maize. Science, 326, 1115–1117. 10.1126/science.1177837 19965431

[tpg220046-bib-0027] Hadjiargyrou, M. , & Delihas, N. (2013). The intertwining of transposable elements and non‐coding RNAs. International Journal of Molecular Sciences, 14, 13307–13328. 10.3390/ijms140713307 23803660 PMC3742188

[tpg220046-bib-0028] Hawkins, J. S. , Kim, H. , Nason, J. D. , Wing, R. A. , & Wendel, J. F. (2006). Differential lineage‐specific amplification of transposable elements is responsible for genome size variation in *Gossypium* . Genome Research, 16, 1252–1261. 10.1101/gr.5282906 16954538 PMC1581434

[tpg220046-bib-0029] Hirsch, C. N. , Foerster, J. M. , Johnson, J. M. , Sekhon, R. S. , Muttoni, G. , Vaillancourt, B. , … Buell, C. R. (2014). Insights into the maize pan‐genome and pan‐transcriptome. Plant Cell, 26, 121–135. 10.1105/tpc.113.119982 24488960 PMC3963563

[tpg220046-bib-0030] Hou, J. , Lu, D. , Mason, A. S. , Li, B. , Xiao, M. , An, S. , & Fu, D. (2019). Non‐coding RNAs and transposable elements in plant genomes: Emergence, regulatory mechanisms and roles in plant development and stress responses. Planta, 250, 23–40. 10.1007/s00425-019-03166-7 30993403

[tpg220046-bib-0031] Hu, Z. , Sun, C. , Lu, K. C. , Chu, X. , Zhao, Y. , Lu, J. , … Wei, C. (2017). EUPAN enables pan‐genome studies of a large number of eukaryotic genomes. Bioinformatics, 33, 2408–2409. 10.1093/bioinformatics/btx170 28369371

[tpg220046-bib-0032] Hubner, S. , Bercovich, N. , Todesco, M. , Mandel, J. R. , Odenheimer, J. , Ziegler, E. , … Rieseberg, L. H. (2019). Sunflower pan‐genome analysis shows that hybridization altered gene content and disease resistance. Nature Plants, 5, 54–62. 10.1038/s41477-018-0329-0 30598532

[tpg220046-bib-0033] Hurgobin, B. , & Edwards, D. (2017). SNP discovery using a pangenome: Has the single reference approach become obsolete? Biology, 6, 21. 10.3390/biology6010021 28287462 PMC5372014

[tpg220046-bib-0034] Hurgobin, B. , Golicz, A. A. , Bayer, P. E. , Chan, C. K. K. , Tirnaz, S. , Dolatabadian, A. , … Edwards, D. (2018). Homoeologous exchange is a major cause of gene presence/absence variation in the amphidiploid *Brassica napus* . Plant Biotechnology Journal, 16, 1265–1274. 10.1111/pbi.12867 29205771 PMC5999312

[tpg220046-bib-0035] Iqbal, Z. , Caccamo, M. , Turner, I. , Flicek, P. , & McVean, G. (2012). De novo assembly and genotyping of variants using colored de Bruijn graphs. Nature Genetics, 44, 226–232. 10.1038/ng.1028 22231483 PMC3272472

[tpg220046-bib-0036] Kapusta, A. , Kronenberg, Z. , Lynch, V. J. , Zhuo, X. , Ramsay, L. , Bourque, G. , … Feschotte, C. (2013). Transposable elements are major contributors to the origin, diversification, and regulation of vertebrate long noncoding RNAs. PLoS Genetics, 9, e1003470. 10.1371/journal.pgen.1003470 23637635 PMC3636048

[tpg220046-bib-0037] Kashkush, K. , Feldman, M. , & Levy, A. A. (2002). Transcriptional activation of retrotransposons alters the expression of adjacent genes in wheat. Nature Genetics, 33, 102. 10.1038/ng1063 12483211

[tpg220046-bib-0038] Kelley, D. , & Rinn, J. (2012). Transposable elements reveal a stem cell‐specific class of long noncoding RNAs. Genome Biology, 13, R107. 10.1186/gb-2012-13-11-r107 23181609 PMC3580499

[tpg220046-bib-0039] Khan, A. W. , Garg, V. , Roorkiwal, M. , Golicz, A. A. , Edwards, D. , & Varshney, R. K. (2019). Super‐pangenome by integrating the wild side of a species for accelerated crop improvement. Trends in Plant Sciences, 25, 148–158. 10.1016/j.tplants.2019.10.012 PMC6988109 31787539

[tpg220046-bib-0040] Lai, K. , Lorenc, M. T. , Lee, H. C. , Berkman, P. J. , Bayer, P. E. , Visendi, P. , … Chan, C. K. K. (2015). Identification and characterization of more than 4 million intervarietal SNP s across the group 7 chromosomes of bread wheat. Plant Biotechnology Journal, 13, 97–104. 10.1111/pbi.12240 25147022

[tpg220046-bib-0041] Li, L. , Stoeckert, C. J., Jr. , & Roos, D. S. (2003). OrthoMCL: Identification of ortholog groups for eukaryotic genomes. Genome Research, 13, 2178–2189. 10.1101/gr.1224503 12952885 PMC403725

[tpg220046-bib-0042] Li, S. F. , Vandivier, L. E. , Tu, B. , Gao, L. , Won, S. Y. , Li, S. B. , … Chen, X. M. (2015). Detection of Pol IV/RDR2‐dependent transcripts at the genomic scale in *Arabidopsis* reveals features and regulation of siRNA biogenesis. Genome Research, 25, 235–245. 10.1101/gr.182238.114 25414514 PMC4315297

[tpg220046-bib-0043] Li, Y. , Li, C. Q. , Xia, J. , & Jin, Y. X. (2011). Domestication of transposable elements into microRNA genes in plants. PLoS ONE, 6, e19212. 10.1371/journal.pone.0019212 21559273 PMC3086885

[tpg220046-bib-0044] Li, Y. H. , Zhou, G. , Ma, J. , Jiang, W. , Jin, L. G. , Zhang, Z. , … Qiu, L. J. (2014). De novo assembly of soybean wild relatives for pan‐genome analysis of diversity and agronomic traits. Nature Biotechnology, 32, 1045–1052. 10.1038/nbt.2979 25218520

[tpg220046-bib-0045] Liang, D. , & Wilusz, J. E. (2014). Short intronic repeat sequences facilitate circular RNA production. Genes & Development, 28, 2233–2247. 10.1101/gad.251926.114 25281217 PMC4201285

[tpg220046-bib-0046] Lin, K. , Zhang, N. , Severing, E. I. , Nijveen, H. , Cheng, F. , Visser, R. G. , … Bonnema, G. (2014). Beyond genomic variation–comparison and functional annotation of three *Brassica rapa* genomes: A turnip, a rapid cycling and a Chinese cabbage. BMC Genomics, 15, 250. 10.1186/1471-2164-15-250 24684742 PMC4230417

[tpg220046-bib-0047] Liu, J. , Jung, C. , Xu, J. , Wang, H. , Deng, S. , Bernad, L. , … Chua, N. H. (2012). Genome‐wide analysis uncovers regulation of long intergenic noncoding RNAs in *Arabidopsis* . Plant Cell, 24, 4333–4345. 10.1105/tpc.112.102855 23136377 PMC3531837

[tpg220046-bib-0048] Liu, Y. , Tahir ul Qamar, M. , Feng, J. W. , Ding, Y. , Wang, S. , Wu, G. , … Chen, L. L. (2019). Comparative analysis of miniature inverted–repeat transposable elements (MITEs) and long terminal repeat (LTR) retrotransposons in six Citrus species. BMC Plant Biology, 19, 140. 10.1186/s12870-019-1757-3 30987586 PMC6466647

[tpg220046-bib-0049] Lorenzetti, A. P. R. , de Antonio, G. Y. A. , Paschoal, A. R. , & Domingues, D. S. (2016). PlanTE‐MIR DB: A database for transposable element‐related microRNAs in plant genomes. Functional & Integrative Genomics, 16, 235–242. 10.1007/s10142-016-0480-5 26887375

[tpg220046-bib-0050] Lu, C. , Chen, J. , Zhang, Y. , Hu, Q. , Su, W. , & Kuang, H. (2011). Miniature inverted–repeat transposable elements (MITEs) have been accumulated through amplification bursts and play important roles in gene expression and species diversity in *Oryza sativa* . Molecular Biology and Evolution, 29, 1005–1017. 10.1093/molbev/msr282 22096216 PMC3278479

[tpg220046-bib-0051] Lu, F. , Romay, M. C. , Glaubitz, J. C. , Bradbury, P. J. , Elshire, R. J. , Wang, T. , … Buckler, E. S. (2015). High‐resolution genetic mapping of maize pan‐genome sequence anchors. Nature Communications, 6, 6914. 10.1038/ncomms7914 PMC4411285 25881062

[tpg220046-bib-0052] Marcus, S. , Lee, H. , & Schatz, M. C. (2014). SplitMEM: A graphical algorithm for pan‐genome analysis with suffix skips. Bioinformatics, 30, 3476–3483. 10.1093/bioinformatics/btu756 25398610 PMC4253837

[tpg220046-bib-0053] Martinez, G. , Wolff, P. , Wang, Z. , Moreno‐Romero, J. , Santos‐Gonzalez, J. , Conze, L. L. , … Kohler, C. (2018). Paternal easiRNAs regulate parental genome dosage in Arabidopsis. Nature Genetics, 50, 193–198. 10.1038/s41588-017-0033-4 29335548

[tpg220046-bib-0054] Matzke, M. A. , & Mosher, R. A. (2014). RNA‐directed DNA methylation: An epigenetic pathway of increasing complexity. Nature Reviews Genetics, 15, 394–408. 10.1038/nrg3683 24805120

[tpg220046-bib-0055] McNally, K. L. , Childs, K. L. , Bohnert, R. , Davidson, R. M. , Zhao, K. , Ulat, V. J. , … Leach, J. E. (2009). Genomewide SNP variation reveals relationships among landraces and modern varieties of rice. Proceedings of the National Academy of Sciences of the United States of America, 106, 12273–12278. 10.1073/pnas.0900992106 19597147 PMC2718348

[tpg220046-bib-0056] Medini, D. , Donati, C. , Tettelin, H. , Masignani, V. , & Rappuoli, R. (2005). The microbial pan‐genome. Current Opinion in Genetics and Development, 15, 589–594. 10.1016/j.gde.2005.09.006 16185861

[tpg220046-bib-0057] Monat, C. , Tranchand, C. , Engelen, S. , Labadie, K. , Paradis, E. , Tando, N. , & Sabot, F. (2018). Comparison of two African rice species through a new pan‐genomic approach on massive data. bioRxiv, 245431. 10.1101/245431

[tpg220046-bib-0058] Montenegro, J. D. , Golicz, A. A. , Bayer, P. E. , Hurgobin, B. , Lee, H. , Chan, C. K. , … Edwards, D. (2017). The pangenome of hexaploid bread wheat. The Plant Journal, 90, 1007–1013. 10.1111/tpj.13515 28231383

[tpg220046-bib-0059] Morgante, M. , De Paoli, E. , & Radovic, S. (2007). Transposable elements and the plant pan‐genomes. Current Opinion in Plant Biology, 10, 149–155. 10.1016/j.pbi.2007.02.001 17300983

[tpg220046-bib-0060] Ou, L. J. , Li, D. , Lv, J. H. , Chen, W. C. , Zhang, Z. Q. , Li, X. F. , … Zou, X. X. (2018). Pan‐genome of cultivated pepper (*Capsicum*) and its use in gene presence‐absence variation analyses. New Phytologist, 220, 360–363. 10.1111/nph.15413 30129229

[tpg220046-bib-0061] Paytuví Gallart, A. , Hermoso Pulido, A. , Anzar Martínez de Lagrán, I. , Sanseverino, W. , & Aiese Cigliano, R. (2015). GREENC: A Wiki‐based database of plant lncRNAs. Nucleic Acids Research, 44, D1161–D1166. 10.1093/nar/gkv1215 26578586 PMC4702861

[tpg220046-bib-0062] Pinosio, S. , Giacomello, S. , Faivre‐Rampant, P. , Taylor, G. , Jorge, V. , Le Paslier, M. C. , … Morgante, M. (2016). Characterization of the poplar pan‐genome by genome‐wide identification of structural variation. Molecular Biology and Evolution, 33, 2706–2719. 10.1093/molbev/msw161 27499133 PMC5026262

[tpg220046-bib-0063] Piriyapongsa, J. , & Jordan, I. K. (2008). Dual coding of siRNAs and miRNAs by plant transposable elements. RNA, 14, 814–821. 10.1261/rna.916708 18367716 PMC2327354

[tpg220046-bib-0064] Piriyapongsa, J. , Marino‐Ramirez, L. , & Jordan, I. K. (2007). Origin and evolution of human microRNAs from transposable elements. Genetics, 176, 1323–1337. 10.1534/genetics.107.072553 17435244 PMC1894593

[tpg220046-bib-0065] Plissonneau, C. , Hartmann, F. E. , & Croll, D. (2018). Pangenome analyses of the wheat pathogen *Zymoseptoria tritici* reveal the structural basis of a highly plastic eukaryotic genome. BMC Biology, 16, 5. 10.1186/s12915-017-0457-4 29325559 PMC5765654

[tpg220046-bib-0066] Rasko, D. A. , Rosovitz, M. J. , Myers, G. S. , Mongodin, E. F. , Fricke, W. F. , Gajer, P. , … Ravel, J. (2008). The pangenome structure of *Escherichia coli*: Comparative genomic analysis of *E. coli* commensal and pathogenic isolates. Journal of Bacteriology, 190, 6881–6893. 10.1128/JB.00619-08 18676672 PMC2566221

[tpg220046-bib-0067] Redon, R. , Ishikawa, S. , Fitch, K. R. , Feuk, L. , Perry, G. H. , Andrews, T. D. , … Hurles, M. E. (2006). Global variation in copy number in the human genome. Nature, 444, 444–454. 10.1038/nature05329 17122850 PMC2669898

[tpg220046-bib-0068] Sampath, P. , Murukarthick, J. , Izzah, N. K. , Lee, J. , Choi, H. I. , Shirasawa, K. , … Yang, T. J. (2014). Genome‐wide comparative analysis of 20 miniature inverted‐repeat transposable element families in *Brassica rapa* and *B. oleracea* . PLoS ONE, 9, e94499. 10.1371/journal.pone.0094499 24747717 PMC3991616

[tpg220046-bib-0069] Saxena, R. K. , Edwards, D. , & Varshney, R. K. (2014). Structural variations in plant genomes. Briefings in Functional Genomics, 13, 296–307. 10.1093/bfgp/elu016 24907366 PMC4110416

[tpg220046-bib-0070] Schatz, M. C. , Maron, L. G. , Stein, J. C. , Wences, A. , Gurtowski, J. , Biggers, E. , … McCombie, W. R. (2014). Whole genome *de novo* assemblies of three divergent strains of rice, *Oryza sativa*, document novel gene space of *aus* and *indica* . Genome Biology, 15, 506. 10.1186/PREACCEPT-2784872521277375 25468217 PMC4268812

[tpg220046-bib-0071] Scherer, S. W. , Lee, C. , Birney, E. , Altshuler, D. M. , Eichler, E. E. , Carter, N. P. , … Feuk, L. (2007). Challenges and standards in integrating surveys of structural variation. Nature Genetics, 39, S7–S15. 10.1038/ng2093 17597783 PMC2698291

[tpg220046-bib-0072] Schmid, K. J. , Ramos‐Onsins, S. , Ringys‐Beckstein, H. , Weisshaar, B. , & Mitchell‐Olds, T. (2005). A multilocus sequence survey in *Arabidopsis thaliana* reveals a genome‐wide departure from a neutral model of DNA sequence polymorphism. Genetics, 169, 1601–1615. 10.1534/genetics.104.033795 15654111 PMC1449538

[tpg220046-bib-0073] Schramke, V. , & Allshire, R. (2003). Hairpin RNAs and retrotransposon LTRs effect RNAi and chromatin‐based gene silencing. Science, 301, 1069–1074. 10.1126/science.1086870 12869699

[tpg220046-bib-0074] Segerman, B. (2012). The genetic integrity of bacterial species: The core genome and the accessory genome, two different stories. Frontiers in Cellular and Infection Microbiology, 2, 116. 10.3389/fcimb.2012.00116 22973561 PMC3434323

[tpg220046-bib-0075] Sherman, R. M. , & Salzberg, S. L. (2020). Pan‐genomics in the human genome era. Nature Reviews Genetics, 21, 243–253. 10.1038/s41576-020-0210-7 PMC7752153 32034321

[tpg220046-bib-0076] Snipen, L. , Almoy, T. , & Ussery, D. W. (2009). Microbial comparative pan‐genomics using binomial mixture models. BMC Genomics, 10, 385. 10.1186/1471-2164-10-385 19691844 PMC2907702

[tpg220046-bib-0077] Song, J. M. , Guan, Z. , Hu, J. , Guo, C. , Yang, Z. , Wang, S. , … Zhou, R. J. N. P. (2020). Eight high‐quality genomes reveal pan‐genome architecture and ecotype differentiation of *Brassica napus* . Nature Plants, 6, 34–45. 10.1038/s41477-019-0577-7 31932676 PMC6965005

[tpg220046-bib-0078] Sun, C. , Hu, Z. , Zheng, T. , Lu, K. , Zhao, Y. , Wang, W. , … Zhang, D. (2016). RPAN: Rice pan‐genome browser for ∼3000 rice genomes. Nucleic Acids Research, 45, 597–605. 10.1093/2Fnar/2Fgkw958 27940610 PMC5314802

[tpg220046-bib-0079] Sun, L. , Jing, Y. , Liu, X. , Li, Q. , Xue, Z. , Cheng, Z. , … Qian, W. (2020). Heat stress‐induced transposon activation correlates with 3D chromatin organization rearrangement in *Arabidopsis* . Nature Communications, 11, 1–13. 10.1038/s41467-020-15809-5 PMC7170881 32312999

[tpg220046-bib-0080] Tahir Ul Qamar, M. , Zhu, X. , Xing, F. , & Chen, L. L. (2019). ppsPCP: A plant presence/absence variants scanner and pan‐genome construction pipeline. Bioinformatics, 35, 4156–4158. 10.1093/bioinformatics/btz168 30851098

[tpg220046-bib-0081] Tanzer, A. , & Stadler, P. F. (2004). Molecular evolution of a microRNA cluster. Journal of Molecular Biology, 339, 327–335. 10.1016/j.jmb.2004.03.065 15136036

[tpg220046-bib-0082] Tao, Y. , Zhao, X. , Mace, E. , Henry, R. , & Jordan, D. (2018). Exploring and exploiting pan‐genomics for crop improvement. Molecular Plant, 12, 156–169. 10.1016/j.molp.2018.12.016 30594655

[tpg220046-bib-0083] Tarailo‐Graovac, M. , & Chen, N. (2009). Using RepeatMasker to identify repetitive elements in genomic sequences. Current Protocols Bioinformatics, 25, 1–14. 10.1002/0471250953.bi0410s25 19274634

[tpg220046-bib-0084] Tenaillon, M. I. , Hollister, J. D. , & Gaut, B. S. (2010). A triptych of the evolution of plant transposable elements. Trends in Plant Science, 15, 471–478. 10.1016/j.tplants.2010.05.003 20541961

[tpg220046-bib-0085] Tettelin, H. , Masignani, V. , Cieslewicz, M. J. , Donati, C. , Medini, D. , Ward, N. L. , … Durkin, A. S. (2005). Genome analysis of multiple pathogenic isolates of *Streptococcus agalactiae*: Implications for the microbial “pan‐genome”. Proceedings of the National Academy of Sciences of the United States of America, 102, 13950–13955. 10.1073/pnas.0506758102 16172379 PMC1216834

[tpg220046-bib-0086] Tetz, V. V. (2005). The pangenome concept: A unifying view of genetic information. Medical Science Monitor, 11, HY24–HY29. Retrieved from https://www.ncbi.nlm.nih.gov/pubmed/15990697 15990697

[tpg220046-bib-0087] Tranchant‐Dubreuil, C. , Rouard, M. , & Sabot, F. (2019). Plant pangenome: Impacts on phenotypes and evolution. Annual Plant Reviews, 2. 10.1002/9781119312994.apr0664

[tpg220046-bib-0088] Vernikos, G. , Medini, D. , Riley, D. R. , & Tettelin, H. (2015). Ten years of pan‐genome analyses. Current Opinion in Microbiology, 23, 148–154. 10.1016/j.mib.2014.11.016 25483351

[tpg220046-bib-0089] Voinnet, O. (2009). Origin, biogenesis, and activity of plant microRNAs. Cell, 136, 669–687. 10.1016/j.cell.2009.01.046 19239888

[tpg220046-bib-0090] Wang, D. , Qu, Z. , Yang, L. , Zhang, Q. , Liu, Z. H. , Do, T. , … Zhu, J. K. (2017). Transposable elements (TE s) contribute to stress‐related long intergenic noncoding RNA s in plants. The Plant Journal, 90, 133–146. 10.1111/tpj.13481 28106309 PMC5514416

[tpg220046-bib-0091] Wang, W. , Mauleon, R. , Hu, Z. , Chebotarov, D. , Tai, S. , Wu, Z. , … Leung, H. (2018). Genomic variation in 3,010 diverse accessions of Asian cultivated rice. Nature, 557, 43–49. 10.1038/s41586-018-0063-9 29695866 PMC6784863

[tpg220046-bib-0092] Wang, Z. P. , Liu, Y. F. , Li, D. W. , Li, L. , Zhang, Q. , Wang, S. B. , & Huang, H. W. (2017). Identification of circular RNAs in kiwifruit and their species‐specific response to bacterial canker pathogen invasion. Frontiers in Plant Science, 8, 413. 10.3389/fpls.2017.00413 28396678 PMC5366334

[tpg220046-bib-0093] Wei, J. W. , Huang, K. , Yang, C. , & Kang, C. S. (2017). Non‐coding RNAs as regulators in epigenetics. Oncology Reports, 37, 3–9. 10.3892/or.2016.5236 27841002

[tpg220046-bib-0094] Wendel, J. F. , Jackson, S. A. , Meyers, B. C. , & Wing, R. A. (2016). Evolution of plant genome architecture. Genome Biology, 17, 37. 10.1186/s13059-016-0908-1 26926526 PMC4772531

[tpg220046-bib-0095] Wicker, T. , Sabot, F. , Hua‐Van, A. , Bennetzen, J. L. , Capy, P. , Chalhoub, B. , … Schulman, A. H. (2007). A unified classification system for eukaryotic transposable elements. Nature Reviews Genetics, 8, 973–982. 10.1038/nrg2165 17984973

[tpg220046-bib-0096] Wierzbicki, A. T. , Haag, J. R. , & Pikaard, C. S. (2008). Noncoding transcription by RNA polymerase Pol IVb/Pol V mediates transcriptional silencing of overlapping and adjacent genes. Cell, 135, 635–648. 10.1016/j.cell.2008.09.035 19013275 PMC2602798

[tpg220046-bib-0097] Xiao, H. , Jiang, N. , Schaffner, E. , Stockinger, E. J. , & van der Knaap, E. (2008). A retrotransposon‐mediated gene duplication underlies morphological variation of tomato fruit. Science, 319, 1527–1530. 10.1126/science.1153040 18339939

[tpg220046-bib-0098] Xiao, J. , Zhang, Z. , Wu, J. , & Yu, J. (2015). A brief review of software tools for pangenomics. Genomics Proteomics Bioinformatics, 13, 73–76. 10.1016/j.gpb.2015.01.007 25721608 PMC4411478

[tpg220046-bib-0099] Yao, W. , Li, G. W. , Zhao, H. , Wang, G. W. , Lian, X. M. , & Xie, W. B. (2015). Exploring the rice dispensable genome using a metagenome‐like assembly strategy. Genome Biology, 16, 187. 10.1186/s13059-015-0757-3 26403182 PMC4583175

[tpg220046-bib-0100] Yi, X. , Zhang, Z. , Ling, Y. , Xu, W. , & Su, Z. (2014). PNRD: A plant non‐coding RNA database. Nucleic Acids Research, 43, D982–D989. 10.1093/nar/gku1162 25398903 PMC4383960

[tpg220046-bib-0101] Yu, J. , Golicz, A. A. , Lu, K. , Dossa, K. , Zhang, Y. , Chen, J. , … Edwards, D. (2019). Insight into the evolution and functional characteristics of the pan‐genome assembly from sesame landraces and modern cultivars. Plant Biotechnology Journal, 17, 881–892. 10.1111/pbi.13022 30315621 PMC6587448

[tpg220046-bib-0102] Zerjal, T. , Joets, J. , Alix, K. , Grandbastien, M. A. , & Tenaillon, M. I. (2009). Contrasting evolutionary patterns and target specificities among three Tourist‐like MITE families in the maize genome. Plant Molecular Biology, 71, 99–114. 10.1007/s11103-009-9511-0 19533380

[tpg220046-bib-0103] Zhai, J. , Bischof, S. , Wang, H. , Feng, S. , Lee, T. F. , Teng, C. , … Jacobsen, S. E. (2015). A one precursor one siRNA model for pol IV‐dependent siRNA biogenesis. Cell, 163, 445–455. 10.1016/j.cell.2015.09.032 26451488 PMC5023148

[tpg220046-bib-0104] Zhang, D. X. , & Hewitt, G. M. (2003). Nuclear DNA analyses in genetic studies of populations: Practice, problems and prospects. Molecular Ecology, 12, 563–584. 10.1046/j.1365-294x.2003.01773.x 12675814

[tpg220046-bib-0105] Zhang, H. T. , Tao, Z. , Hong, H. M. , Chen, Z. H. , Wu, C. Y. , Li, X. H. , … Wang, S. P. (2016). Transposon‐derived small RNA is responsible for modified function of WRKY45 locus. Nature Plants, 2, 16016. 10.1038/Nplants.2016.16 27249351

[tpg220046-bib-0106] Zhang, Y. , Jiang, W. K. , & Gao, L. Z. (2011). Evolution of microRNA genes in *Oryza sativa* and *Arabidopsis thaliana*: An update of the inverted duplication model. PLoS ONE, 6, e28073. 10.1371/journal.pone.0028073 22194805 PMC3237417

[tpg220046-bib-0107] Zhao, J. , Bayer, P. E. , Ruperao, P. , Saxena, R. K. , Khan, A. W. , … Varshney, R. K. (2020). Trait associations in the pangenome of pigeon pea (*Cajanus cajan*). Plant Biotechnol Journal, 10.1111/pbi.13354 (in press) PMC7415775 32020732

[tpg220046-bib-0108] Zhao, Q. , Feng, Q. , Lu, H. , Li, Y. , Wang, A. , Tian, Q. , … Huang, T. (2018). Pan‐genome analysis highlights the extent of genomic variation in cultivated and wild rice. Nature Genetics, 50, 278. 10.1038/s41588-018-0041-z 29335547

[tpg220046-bib-0109] Zhao, W. , Chu, S. , & Jiao, Y. (2019). Present scenario of circular RNAs (circRNAs) in plants. Frontiers in Plant Science, 10, 379. 10.3389/fpls.2019.00379 31001302 PMC6454147

[tpg220046-bib-0110] Zhong, X. , Du, J. , Hale, C. J. , Gallego‐Bartolome, J. , Feng, S. , Vashisht, A. A. , … Jacobsen, S. E. (2014). Molecular mechanism of action of plant DRM de novo DNA methyltransferases. Cell, 157, 1050–1060. 10.1016/j.cell.2014.03.056 24855943 PMC4123750

[tpg220046-bib-0111] Zhou, P. , Silverstein, K. A. , Ramaraj, T. , Guhlin, J. , Denny, R. , Liu, J. , … Young, N. D. (2017). Exploring structural variation and gene family architecture with *De Novo* assemblies of 15 *Medicago* genomes. BMC Genomics, 18, 261. 10.1186/s12864-017-3654-1 28347275 PMC5369179

