# Supplementary material for: Pan‐genome: A promising resource for noncoding RNA discovery in plants
Source: Plant Genome. 2020 Aug 28;13(3):e20046. doi: 10.1002/tpg2.20046 (PMC12806977; doi:10.1002/tpg2.20046)
Supplement: Supplementary file 1 [file TPG2-13-e20046-s001.docx]

**Figure S1.** Illustration of the major types of sequence variations in plants responsible for their genomic and phenotypic diversity. (**A**) Presence/absence variations PAVs; (**B**) copy number variations (CNVs). Colored blocks represent genes.

**Figure S2.** General representation of different portions and types of pan-genomes. (**A)** A flower-plot illustration representing the core genome, variable genome, and member-specific genome. (**B)** Illustration of open and closed pan-genomes.

**Table S1**. Examples of presence/absence variations (PAVs) and copy number variations (CNVs) and their impact on important agronomic traits of major crops (table modified from (Tao et al., 2018))

| Species | Type | Trait | Genes/Locus | Reference |
| --- | --- | --- | --- | --- |
| Maize | PAVs | Kernel row number; Photoperiod sensitivity; Apical dominance; Flowering time; Resistance to head smut | *KRN4; ZmCCT9; TB1; Vgt1; qHSR1* | (Huang et al., 2018; Liu et al., 2015; Salvi et al., 2007; Studer et al., 2011; Zuo et al., 2015) |
|  | CNVs | Aluminum tolerance | *MATE1* | (Maron et al., 2013) |
| Rice | PAVs | Grain size; Blast resistance; Submergence tolerance; Phosphorus-starvation tolerance; Deep water response; Plant architecture; Blast disease; Hybrid male sterility; Shattering | *GW5/qSW5/GSE5; Pikm1-TS/Pikm2-TS; Sub1A; Pup1; SNORKEL1/SNORKEL2; qPE9-1; Pi21; DPL1/DPL2/S27/S28; OsSh1* | (Ashikawa et al., 2008; Duan et al., 2017; Fukuoka et al., 2009; Gamuyao et al., 2012; Hattori et al., 2009; Lin et al., 2012; Liu et al., 2017; Mizuta et al., 2010; Shomura et al., 2008; Xu et al., 2006; Yamagata et al., 2010; Zhou et al., 2009) |
|  | CNVs | Grain size, grain number, yield; Hybrid male sterility | *GL7/SGDP7; Sc* | (Bai et al., 2017; Shen et al., 2017; Wang et al., 2015) |
| Wheat | PAVs | Leaf rust; Stripe rust; Tan spot and *Stagonospora nodorum* blotch | *Lr10; Yr36; Tsn1* | (Faris et al., 2010; Feuillet et al., 2003; Fu et al., 2009) |
|  | CNVs | Cold tolerance; Vernalization; Photoperiod sensitivity; Plant height | *FR-2; Vrn-A1; Ppd-B1; Rht-D1b* | (Knox et al., 2010; Li et al., 2012; Wurschum et al., 2015) |
| Sorghum | PAVs | Resistance to *Striga*; Shattering; Aluminum tolerance | *LGS1; Sh1; SbMATE* | (Gobena et al., 2017; Lin et al., 2012; Magalhaes et al., 2007) |
| Barley | CNVs | Frost resistance; Boron-toxicity tolerance; Flowering time | *FR-H2; Bot1; HvFT1* | (Francia et al., 2016; Nitcher et al., 2013; Sutton et al., 2007) |
| Potato | PAVs | Resistance against late blight | *R1/ELR* | (Ballvora et al., 2002; Du et al., 2015) |
| Soybean | PAVs | Salt tolerance | *GmCHX1* | (Qi et al., 2014) |
|  | CNVs | Resistance to cyst nematode | *Rhg1* | (Cook et al., 2012) |
| Tomato | PAVs | Flowering | *TMF* | (MacAlister et al., 2012) |
|  | CNVs | Elongated fruit shape | *SUN* | (Xiao et al., 2008) |
| Cucumber | PAVs | Tuberculate fruit | *Tu* | (Yang et al., 2014) |
|  | CNVs | Sexual production | *F* | (Zhang et al., 2015) |

**Table S2.** Number of genes and transposable elements (TEs) contributed by each *Arabidopsis thaliana* ecotype to the pan-genome.

| Reference | Query | No. of genes | No. of TEs |
| --- | --- | --- | --- |
| Col-0 | Bur-0 | 334,67 | 342,55 |
| Draft-pan-genome 1 | Can-0 | 337,57 | 344,80 |
| Draft-pan-genome 2 | Ct-1 | 339,87 | 348,22 |
| Draft-pan-genome 3 | Edi-0 | 340,78 | 348,68 |
| Draft-pan-genome 4 | Hi-0 | 341,98 | 351,16 |
| Draft-pan-genome 5 | Kn-0 | 342,38 | 351,44 |
| Draft-pan-genome 6 | Ler-0 | 343,10 | 353,47 |
| Draft-pan-genome 7 | Mt-0 | 343,82 | 355,83 |
| Draft-pan-genome 8 | No-0 | 344,24 | 356,40 |
| Draft-pan-genome 9 | Oy-0 | 344,76 | 358,51 |
| Draft-pan-genome 10 | Po-0 | 345,03 | 358,67 |
| Draft-pan-genome 11 | Rsch-4 | 345,39 | 360,09 |
| Draft-pan-genome 12 | Sf-2 | 345,93 | 361,49 |
| Draft-pan-genome 13 | Tsu-0 | 346,67 | 364,08 |
| Draft-pan-genome 14 | Wil-2 | 347,09 | 366,19 |
| Draft-pan-genome 15 | Ws-0 | 347,70 | 368,55 |
| Draft-pan-genome 16 | Wu-0 | 348,21 | 370,36 |
| Draft-pan-genome 17 | Zu-0 | 348,58 | 371,32 |
| Final pan-genome of  *A. thaliana* | | 348,99 | 372,88 |

**References**

Ashikawa, I., Hayashi, N., Yamane, H., Kanamori, H., Wu, J., Matsumoto, T., … Yano, M. (2008). Two adjacent nucleotide-binding site–leucine-rich repeat class genes are required to confer Pikm-specific rice blast resistance. *Genetics, 180*(4), 2267-2276. <https://doi.org/10.1534/genetics.108.095034>

Bai, X., Huang, Y., Hu, Y., Liu, H., Zhang, B., Smaczniak, C., … Xing, Y. (2017). Duplication of an upstream silencer of FZP increases grain yield in rice. *Nature Plants, 3*(11), 885-893. <https://doi.org/10.1038/s41477-017-0042-4>

Ballvora, A., Ercolano, M. R., Weiss, J., Meksem, K., Bormann, C. A., … Gebhardt, C. (2002). The R1 gene for potato resistance to late blight (*Phytophthora infestans*) belongs to the leucine zipper/NBS/LRR class of plant resistance genes. *The* *Plant Journal, 30*(3), 361-371. <https://doi.org/10.1046/j.1365-313x.2001.01292.x>

Cook, D. E., Lee, T. G., Guo, X. L., Melito, S., Wang, K., Bayless, A. M., … Bent, A. F. (2012). Copy Number Variation of Multiple Genes at Rhg1 Mediates Nematode Resistance in Soybean. *Science, 338*(6111), 1206-1209. <https://doi.org/10.1126/science.1228746>

Du, J., Verzaux, E., Chaparro-Garcia, A., Bijsterbosch, G., Keizer, L. C., Zhou, J., … Vleeshouwers, V. G. (2015). Elicitin recognition confers enhanced resistance to Phytophthora infestans in potato. *Nature Plants, 1*(4), 15034. https://doi.org/10.1038/nplants.2015.34

Duan, P., Xu, J., Zeng, D., Zhang, B., Geng, M., Zhang, G., … Li, Y. (2017). Natural Variation in the Promoter of GSE5 Contributes to Grain Size Diversity in Rice. *Molecular Plant, 10*(5), 685-694. <https://doi.org/10.1016/j.molp.2017.03.009>

Faris, J. D., Zhang, Z., Lu, H., Lu, S., Reddy, L., Cloutier, S., … Friesen, T. L. (2010, Jul 27). A unique wheat disease resistance-like gene governs effector-triggered susceptibility to necrotrophic pathogens. *Proceedings of the National Academy of Sciences of the United States of America, 107*(30), 13544-13549. <https://doi.org/10.1073/pnas.1004090107>

Feuillet, C., Travella, S., Stein, N., Albar, L., Nublat, A., & Keller, B. (2003, Dec 9). Map-based isolation of the leaf rust disease resistance gene Lr10 from the hexaploid wheat (*Triticum aestivum* L.) genome. *Proceedings of the National Academy of Sciences of the United States of America, 100*(25), 15253-15258. <https://doi.org/10.1073/pnas.2435133100>

Francia, E., Morcia, C., Pasquariello, M., Mazzamurro, V., Milc, J. A., Rizza, F., … Pecchioni, N. (2016). Copy number variation at the HvCBF4–HvCBF2 genomic segment is a major component of frost resistance in barley. *Plant Molecular Biology, 92*(1-2), 161-175. https://doi.org/10.1007/s11103-016-0505-4

Fu, D., Uauy, C., Distelfeld, A., Blechl, A., Epstein, L., Chen, X., … Dubcovsky, J. (2009). A kinase-START gene confers temperature-dependent resistance to wheat stripe rust. *Science, 323*(5919), 1357-1360. <https://doi.org/10.1126/science.1166289>

Fukuoka, S., Saka, N., Koga, H., Ono, K., Shimizu, T., Ebana, K., … Yano, M. (2009). Loss of function of a proline-containing protein confers durable disease resistance in rice. *Science, 325*(5943), 998-1001. <https://doi.org/10.1126/science.1175550>

Gamuyao, R., Chin, J. H., Pariasca-Tanaka, J., Pesaresi, P., Catausan, S., Dalid, C., … Heuer, S. (2012, Aug 23). The protein kinase Pstol1 from traditional rice confers tolerance of phosphorus deficiency. *Nature, 488*(7412), 535-539. <https://doi.org/10.1038/nature11346>

Gobena, D., Shimels, M., Rich, P. J., Ruyter-Spira, C., Bouwmeester, H., Kanuganti, S., … Ejeta, G. (2017). Mutation in sorghum LOW GERMINATION STIMULANT 1 alters strigolactones and causes Striga resistance. *Proceedings of the National Academy of Sciences of the United States of America, 114*(17), 4471-4476. <https://doi.org/10.1073/pnas.1618965114>

Hattori, Y., Nagai, K., Furukawa, S., Song, X. J., Kawano, R., Sakakibara, H., … Ashikari, M. (2009, Aug 20). The ethylene response factors SNORKEL1 and SNORKEL2 allow rice to adapt to deep water. *Nature, 460*(7258), 1026-1030. <https://doi.org/10.1038/nature08258>

Huang, C., Sun, H., Xu, D., Chen, Q., Liang, Y., Wang, X., … Tian, F. (2018, Jan 9). ZmCCT9 enhances maize adaptation to higher latitudes. *Proceedings of the National Academy of Sciences of the United States of America, 115*(2), E334-E341. <https://doi.org/10.1073/pnas.1718058115>

Knox, A. K., Dhillon, T., Cheng, H. M., Tondelli, A., Pecchioni, N., & Stockinger, E. J. (2010, Jun). CBF gene copy number variation at Frost Resistance-2 is associated with levels of freezing tolerance in temperate-climate cereals. *Theoretical and Applied Genetics, 121*(1), 21-35. <https://doi.org/10.1007/s00122-010-1288-7>

Li, Y., Xiao, J., Wu, J., Duan, J., Liu, Y., Ye, X., … Kong, X. (2012). A tandem segmental duplication (TSD) in green revolution gene Rht-D1b region underlies plant height variation. *New Phytologists, 196*(1), 282-291. <https://doi.org/10.1111/j.1469-8137.2012.04243.x>

Lin, Z., Li, X., Shannon, L. M., Yeh, C. T., Wang, M. L., Bai, G., … Yu, J. (2012). Parallel domestication of the Shattering1 genes in cereals. *Nature Genetics, 44*(6), 720-724. <https://doi.org/10.1038/ng.2281>

Liu, J., Chen, J., Zheng, X., Wu, F., Lin, Q., Heng, Y., … Wan, J. (2017). GW5 acts in the brassinosteroid signalling pathway to regulate grain width and weight in rice. *Nature Plants, 3*(5), 17043. <https://doi.org/10.1038/nplants.2017.43>

Liu, L., Du, Y., Shen, X., Li, M., Sun, W., Huang, J., … Zhang, Z. (2015). KRN4 Controls Quantitative Variation in Maize Kernel Row Number. *PLoS Genetics, 11*(11), e1005670. <https://doi.org/10.1371/journal.pgen.1005670>

MacAlister, C. A., Park, S. J., Jiang, K., Marcel, F., Bendahmane, A., Izkovich, Y., … Lippman, Z. B. (2012). Synchronization of the flowering transition by the tomato TERMINATING FLOWER gene. *Nature Genetics, 44*(12), 1393-1398. <https://doi.org/10.1038/ng.2465>

Magalhaes, J. V., Liu, J., Guimaraes, C. T., Lana, U. G., Alves, V. M., Wang, Y. H., … Kochian, L. V. (2007). A gene in the multidrug and toxic compound extrusion (MATE) family confers aluminum tolerance in sorghum. *Nature Genetics, 39*(9), 1156-1161. <https://doi.org/10.1038/ng2074>

Maron, L. G., Guimaraes, C. T., Kirst, M., Albert, P. S., Birchler, J. A., Bradbury, P. J., … Kochian, L. V. (2013). Aluminum tolerance in maize is associated with higher MATE1 gene copy number. *Proceedings of the National Academy of Sciences of the United States of America, 110*(13), 5241-5246. <https://doi.org/10.1073/pnas.1220766110>

Mizuta, Y., Harushima, Y., & Kurata, N. (2010). Rice pollen hybrid incompatibility caused by reciprocal gene loss of duplicated genes. *Proceedings of the National Academy of Sciences of the United States of America, 107*(47), 20417-20422. <https://doi.org/10.1073/pnas.1003124107>

Nitcher, R., Distelfeld, A., Tan, C., Yan, L. L., & Dubcovsky, J. (2013). Increased copy number at the HvFT1 locus is associated with accelerated flowering time in barley. *Molecular Genetics and Genomics, 288*(5-6), 261-275. <https://doi.org/10.1007/s00438-013-0746-8>

Qi, X., Li, M. W., Xie, M., Liu, X., Ni, M., Shao, G., … Lam, H. M. (2014). Identification of a novel salt tolerance gene in wild soybean by whole-genome sequencing. *Nature Communications, 5*, 4340. <https://doi.org/10.1038/ncomms5340>

Salvi, S., Sponza, G., Morgante, M., Tomes, D., Niu, X., Fengler, K. A., … Tuberosa, R. (2007). Conserved noncoding genomic sequences associated with a flowering-time quantitative trait locus in maize. *Proceedings of the National Academy of Sciences of the United States of America, 104*(27), 11376-11381. <https://doi.org/10.1073/pnas.0704145104>

Shen, R., Wang, L., Liu, X., Wu, J., Jin, W., Zhao, X., … Liu, Y. G. (2017). Genomic structural variation-mediated allelic suppression causes hybrid male sterility in rice. *Nat Communications, 8*(1), 1310. <https://doi.org/10.1038/s41467-017-01400-y>

Shomura, A., Izawa, T., Ebana, K., Ebitani, T., Kanegae, H., Konishi, S., & Yano, M. (2008). Deletion in a gene associated with grain size increased yields during rice domestication. *Nature Genetics, 40*(8), 1023-1028. <https://doi.org/10.1038/ng.169>

Studer, A., Zhao, Q., Ross-Ibarra, J., & Doebley, J. (2011). Identification of a functional transposon insertion in the maize domestication gene tb1. *Nature Genetics, 43*(11), 1160-1163. <https://doi.org/10.1038/ng.942>

Sutton, T., Baumann, U., Hayes, J., Collins, N. C., Shi, B. J., Schnurbusch, T., … Langridge, P. (2007). Boron-toxicity tolerance in barley arising from efflux transporter amplification. *Science, 318*(5855), 1446-1449. <https://doi.org/10.1126/science.1146853>

Tao, Y., Zhao, X., Mace, E., Henry, R., & Jordan, D. (2018). Exploring and exploiting pan-genomics for crop improvement. *Molecular plant, 12*(2), 156-169. <https://doi.org/10.1016/j.molp.2018.12.016>

Wang, Y. X., Xiong, G. S., Hu, J., Jiang, L., Yu, H., Xu, J., … Qian, Q. (2015). Copy number variation at the GL7 locus contributes to grain size diversity in rice. *Nature Genetics, 47*(8), 944-+. <https://doi.org/10.1038/ng.3346>

Wurschum, T., Boeven, P. H. G., Langer, S. M., Longin, C. F. H., & Leiser, W. L. (2015). Multiply to conquer: Copy number variations at Ppd-B1 and Vrn-A1 facilitate global adaptation in wheat. *BMC Genetics, 16*(1), 96. [https://doi.org/](https://doi.org/ARTN)10.1186/s12863-015-0258-0

Xiao, H., Jiang, N., Schaffner, E., Stockinger, E. J., & van der Knaap, E. (2008). A retrotransposon-mediated gene duplication underlies morphological variation of tomato fruit. *Science, 319*(5869), 1527-1530. <https://doi.org/10.1126/science.1153040>

Xu, K., Xu, X., Fukao, T., Canlas, P., Maghirang-Rodriguez, R., Heuer, S., … Mackill, D. J. (2006). Sub1A is an ethylene-response-factor-like gene that confers submergence tolerance to rice. *Nature, 442*(7103), 705-708. <https://doi.org/10.1038/nature04920>

Yamagata, Y., Yamamoto, E., Aya, K., Win, K. T., Doi, K., Sobrizal, Ito, T., … Yoshimura, A. (2010). Mitochondrial gene in the nuclear genome induces reproductive barrier in rice. *Proceedings of the National Academy of Sciences of the United States of America, 107*(4), 1494-1499. <https://doi.org/10.1073/pnas.0908283107>

Yang, X. Q., Zhang, W. W., He, H. L., Nie, J. T., Bie, B. B., … Cai, R. (2014). Tuberculate fruit gene Tu encodes a C2H2 zinc finger protein that is required for the warty fruit phenotype in cucumber (*Cucumis sativus* L.). *The Plant Journal, 78*(6), 1034-1046. <https://doi.org/10.1111/tpj.12531>

Zhang, Z. H., Mao, L. Y., Chen, H. M., Bu, F. J., Li, G. C., Sun, J. J., … Huang, S. W. (2015). Genome-Wide Mapping of Structural Variations Reveals a Copy Number Variant That Determines Reproductive Morphology in Cucumber. *Plant Cell, 27*(6), 1595-1604. <https://doi.org/10.1105/tpc.114.135848>

Zhou, Y., Zhu, J., Li, Z., Yi, C., Liu, J., Zhang, H., … Liang, G. (2009). Deletion in a quantitative trait gene qPE9-1 associated with panicle erectness improves plant architecture during rice domestication. *Genetics, 183*(1), 315-324. <https://doi.org/10.1534/genetics.109.102681>

Zuo, W., Chao, Q., Zhang, N., Ye, J., Tan, G., Li, B., … Xu, M. (2015). A maize wall-associated kinase confers quantitative resistance to head smut. *Nature Genetics, 47*(2), 151-157. <https://doi.org/10.1038/ng.3170>
